# Structure of i-Motif/Duplex Junctions at Neutral pH

**DOI:** 10.1021/jacs.1c04679

**Published:** 2021-08-09

**Authors:** Israel Serrano-Chacón, Bartomeu Mir, Núria Escaja, Carlos González

**Affiliations:** † Instituto de Química Física ‘Rocasolano’, CSIC, Serrano 119, 28006 Madrid, Spain; ‡ Inorganic and Organic Chemistry Department, Organic Chemistry Section, and IBUB, University of Barcelona, Martí i Franquès 1-11, 08028 Barcelona, Spain; § BIOESTRAN associated unit UB-CSIC, 08028 Barcelona, Spain

## Abstract

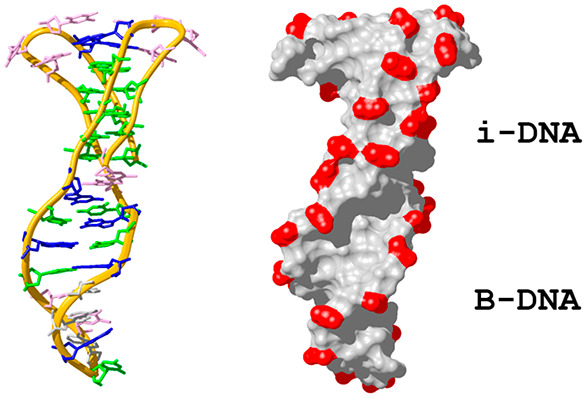

We report here the three-dimensional structure of an i-motif/duplex junction, determined by NMR methods at neutral pH. By including a minor groove tetrad at one side of the C:C^+^ stack of a monomeric i-motif, and a stem/loop hairpin at the other side, we have designed stable DNA constructs in which i-DNA and B-DNA regions coexist in a wide range of experimental conditions. This study demonstrates that i- and B-DNA are structurally compatible, giving rise to a distinctive fold with peculiar groove shapes. The effect of different residues at the i-motif/duplex interface has been explored. We also show that these constructs can be adapted to sequences of biological relevance, like that found in the promoter region of the KRAS oncogene.

The i-motif is a four-stranded intercalated structure stabilized by the formation of hemi-protonated C:C^+^ base-pairs.^1^ Since its recent observation in human cells,^2^ the i-motif (also named as i-DNA)^3^ is attracting extraordinary attention.^4^ Several studies in vitro and in vivo have found i-motifs in biologically relevant C-rich sequences involved in processes like gene transcription^5^ and DNA synthesis.^6^ As in the case of G-quadruplexes, bioinformatics searches indicate that i-motif-forming sequences are common in the genome.^7^ Since DNA is mainly a double helix in the cell, local G-quadruplex or i-motif formation entails the occurrence of interfaces (junctions) with canonical B-form DNA regions. In recent years, G-quadruplex/duplex junctions have been extensively studied^8^ and found to be an interesting target for selective recognition.^9^ However, very little is known about the structure of i-motif/duplex junctions (IDJs), although they are probably as common as those involving G-quadruplexes.^10^

One of the difficulties for studying IDJs is that different experimental conditions are usually required for i-motif and duplex formation. Although some C-rich sequences have been found to form i-motifs at neutral conditions, their stability is usually low. With only one recent exception, all structural studies on i-DNA were carried out at acidic pH.^7^

i-DNA stability follows complex rules still not fully understood. The effects of cytosine tract length and connecting loops have been widely studied in the past few years.^11^ Capping interactions at the sides of the C:C^+^ stack also play important roles in i-motif stability.^12^ In particular, minor groove tetrads (MGTs) have been found to be excellent capping elements, able to stabilize i-motifs at neutral conditions.^7^ MGTs result from the association of Watson–Crick or G:T base-pairs through their minor groove side. A:T:A:T,^13^ A:G:A:G,^14^ G:C:G:C,^13,15^ G:C:G:T, or G:T:G:T minor groove tetrads have been observed in different contexts, and the last three have been found to stabilize i-motifs.^7,16^ The first case of an i-motif stabilized by MGTs was observed in a centromeric sequence stabilized by a G:T:G:T tetrad.^16b^ These tetrads were then found in other dimeric i-motifs,^16a^ and more recently in the structure of the so-called mini i-motif.^7^ The importance of these interactions is reflected by the fact that consensus sequences based on their capability to form favorable C:C^+^/MGT interactions have been found to be prevalent in the human genome, occurring preferentially near regulatory regions.

Following these previous findings, we propose to explore IDJs by designing constructs in which one end of the i-motif is stabilized by a minor groove G:T:G:T tetrad, whereas the other end forms an interface with sequences capable of forming a duplex. For practical reasons, we use a sequence forming a stem-loop hairpin structure, with the stem region being a suitable model of a B-form duplex. The sequence chosen for this region is known to form a stable hairpin with well-dispersed NMR spectra.^17^ A similar strategy has been previously used to build model interfaces between B-DNA and G-quadruplexes.^8a^ These hybrid DNA structures, containing i-motifs and stem/loop hairpins, have been proposed as part of molecular sensors^18^ and most probably occur in biologically relevant sequences in the genome, like those found in promoter regions of KRAS^5a^ or NMYC^19^ oncogenes, as well as in viral genomes.^20^ However, despite their great interest, no detailed structural study in these systems has been published to date.

As a proof of concept, we explored a DNA construct based on the scheme shown in Figure 1A (**IDJ1**). Other related constructs (**IDJ2** and **IDJ3**) are discussed in the Supporting Information (Supplementary Results and Figure S1). The NMR spectra of **IDJ1** exhibit well-dispersed sharp signals (Figure 1C), allowing the identification of the imino resonances corresponding to C:C^+^ (15–16 ppm), Watson–Crick base-pairs (12.5–14.0 ppm), and G:T or T:T mismatches (10.5–12.0 ppm). UV melting curves were recorded at two different pH values (Figure 1B). At pH 7, the melting curve exhibits two clear transitions, at 26.9 and 62.6 °C, whereas at lower pH only a single transition is observed at 59.4 °C. CD spectra at neutral conditions and different temperatures are shown in Figure S2. At low temperature the spectra exhibit a maximum at 284 nm that blue shifts upon heating, and a minimum at 250 nm that red shifts at higher temperature. These experimental data are consistent with the formation of a DNA molecule in which B- and i-DNA regions coexist at neutral pH, as illustrated in Figure 1A. At pH 7, the lower *T*_m_ corresponds to the denaturation of the i-DNA moiety, and the higher *T*_m_ to the denaturation of the B-DNA part.

**Figure 1:**
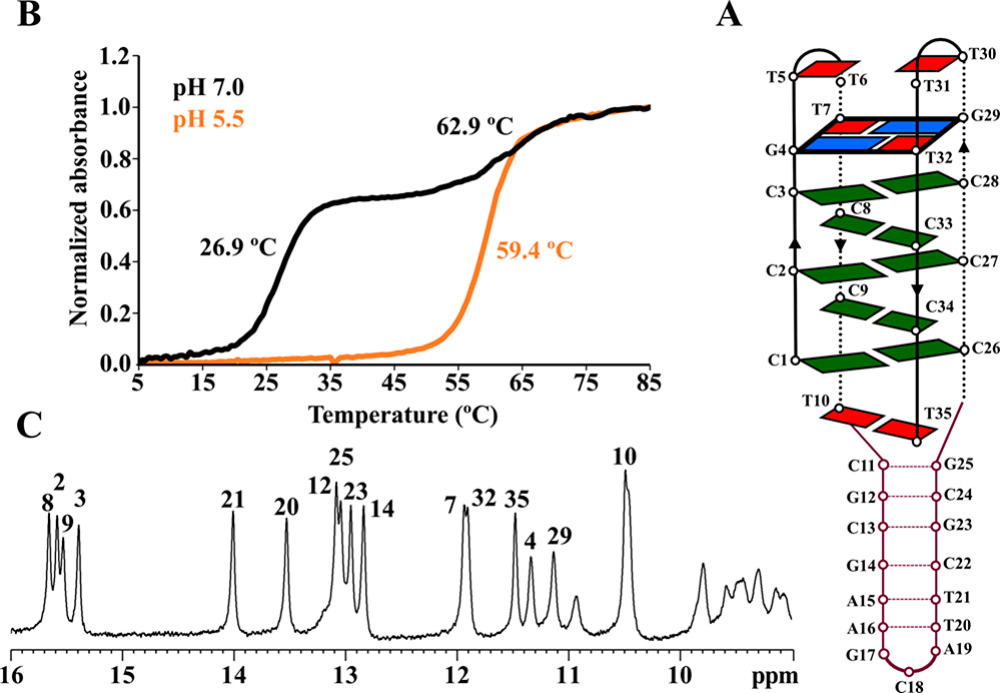
(A) Scheme of **IDJ1**. (B) UV melting curves at pH 5 and 7 ([DNA] = 2 μM). (C) Imino protons region of NMR spectra (pH 7, *T* = 5 °C, [DNA] = 0.5 mM).

First, non-denaturing electrophoretic experiments were performed to rule out the formation of multimeric species (Figure S3). NMR and UV melting data recorded at different DNA concentrations clearly show that the structure is monomeric at conditions adequate for 2D spectra acquisition (Figure S4). Thanks to the good signal dispersion, complete assignment of the NMR spectra of **IDJ1** could be carried out by standard ^1^H NMR methods. The exchangeable protons regions of the NMR spectra are especially informative (Figure 2). Two A:T and four G:C Watson–Crick base-pairs could be easily identified, corresponding to residues in the hairpin stem. NOE sequential connections between these residues could be followed in the sugar/aromatic (Figure S5) and aromatic/aromatic regions. Two G:T base-pairs and one T:T base-pair were detected by their imino–imino and other cross-peaks. Their sequential assignments were performed by analyzing NMR spectra of constructs incorporating ^5m^C, dU, or ^15^N-labeled guanines in key positions (see details in the Supplementary Results and Figures S6–S9). Four cytosine imino signals corresponding to C:C^+^ base-pairs were found. Assignment of the cytosines involved in the i-motif moiety could be done by identifying first the terminal C:C^+^ base-pair adjacent to the G:T:G:T tetrad and then following the characteristic sugar–sugar cross-peaks through the i-motif minor groove. Of particular relevance are the NOEs of T10:T35 with C11:G25 and with cytosines 1 and 26, which indicate the formation of C1:C26^+^, although the corresponding imino proton signal could not be observed. Overall, the spectra are fully consistent with the schematic representation shown in Figure 1. The chemical shifts are listed in Table S2.

**Figure 2:**
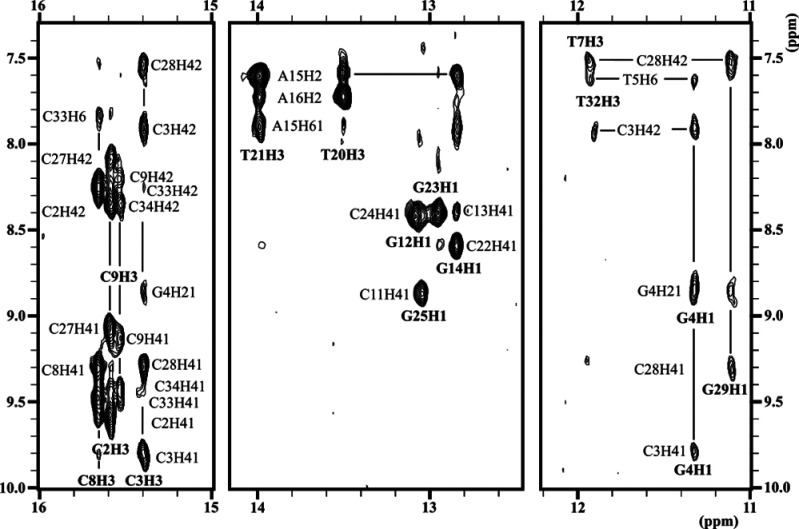
Regions of the NOESY spectra of **IDJ1** (*T* = 5 °C, pH 7, [DNA] = 0.5 mM).

The three-dimensional structure of **IDJ1** was determined on the basis of 238 experimental distance constraints by using restrained molecular dynamics methods (see Supporting Information). Torsion angle constraints were also used for those sugars with a clear south conformation according to J-coupling data (Figure S10). Statistical analysis of distance constraints and the resulting structures are given in Table S3 and Figure S11. Final coordinates are deposited in the PDB (7O5E).

The resulting structure (Figure 3) consists of a stack of five hemi-protonated C:C^+^ base-pairs, surrounded on one side by a minor-groove G:T:G:T tetrad. Two-residue loops connect the residues involved in this tetrad. The first thymine of each loop stacks on top of the tetrad, whereas the other one remains exposed to the solvent. A T:T mismatch is formed at the other end of the C:C^+^ stack, and the structure continues with six Watson–Crick base-pairs without interrupting the base-pair stacking (Figure S12). This stem region adopts a B-form structure with all glycosidic angles in anti and sugars in south conformation. Geometrical parameters are shown in Table S4.

**Figure 3:**
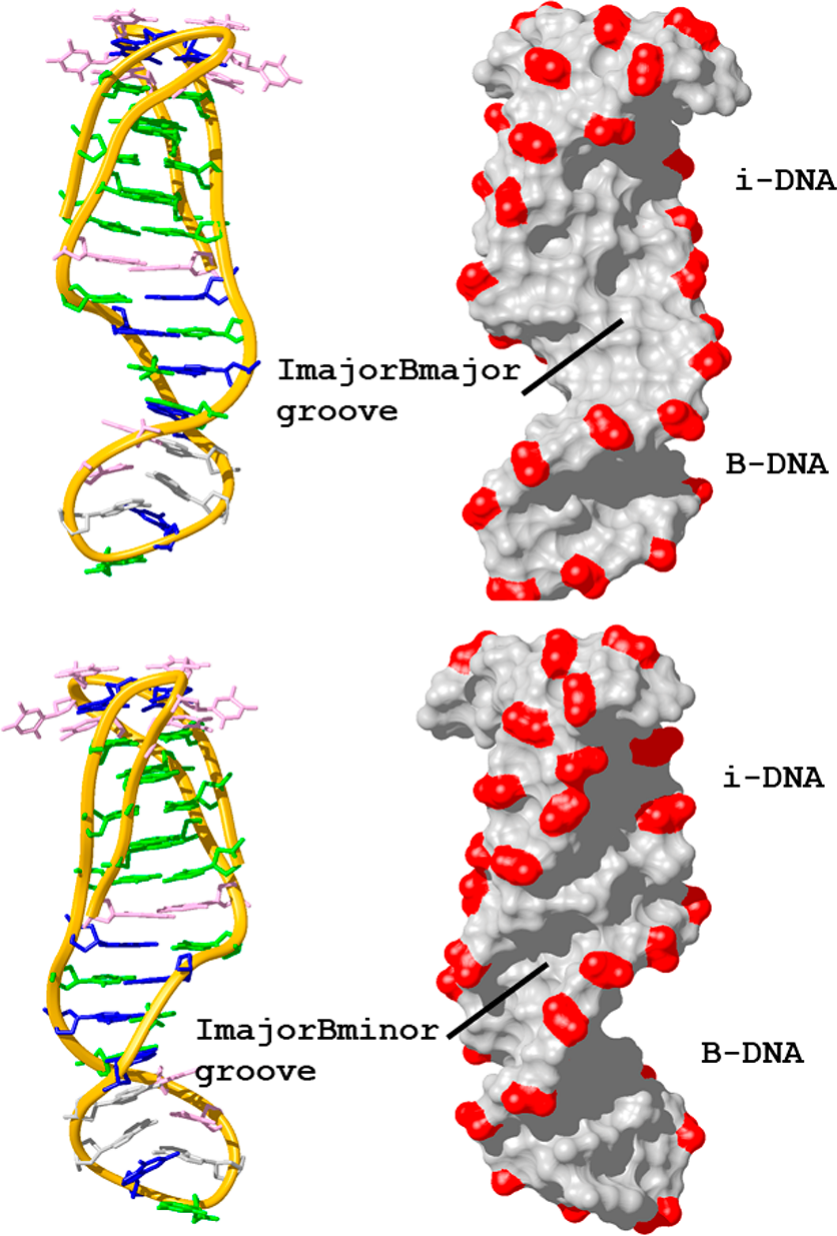
Two views of the **IDJ1** structure showing the resulting characteristic grooves. Phosphate-sugar backbone is shown with a golden ribbon. Phosphates are colored in red in the surface representation. Base color code: cytosines in green, guanines in blue, thymines in pink, and adenines in gray (PDB 7O5E).

The overall molecule’s surface is dominated by two main grooves (Figure 3), resulting from the connection of each of the i-DNA major grooves with the major and minor grooves of the B-DNA region (named as ImajorBmajor and ImajorBminor grooves in the following discussion). Interestingly, the two major grooves in the i-motif region are not identical, as in other i-motifs, as the one connecting with the B-DNA minor groove is narrower than the other (Figure S13). This is most probably a conformational adjustment to the presence of the adjacent B-DNA region. The resulting ImajorBmajor groove exhibits an approximate width of 11–12 Å, being slightly wider in the i-DNA region. On the other hand, the ImajorBminor groove exhibits a width of around 6 Å, also being slightly wider in the i-DNA moiety (Figures 3 and S13). Although the groove widths are similar in the i-motif and duplex regions, their depths are significantly different, being much shallower in the i-DNA.

Details of interface region are shown in Figure 4. The T10:T35 base-pair, formed between parallel oriented strands, continues the intercalation pattern of the C:C^+^ base-pairs in the i-DNA, while interacting with the neighboring G:C base-pair of the B-DNA (Figure 4). The twist angles between these consecutive base-pairs are ∼30° and ∼27°, respectively. The lack of disruption of the base-pair stacking at the junction, together with the fine-tuning of the groove sizes, reflects the compatibility of these two families of DNA structures.

**Figure 4:**
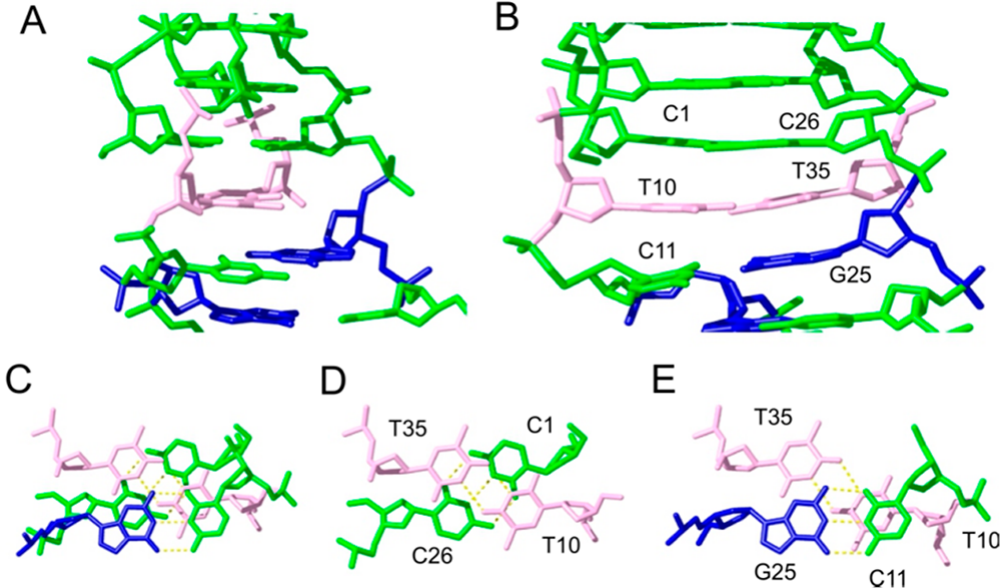
Side and top views of the **IDJ1** interface. Same color code as in Figure 3. Hydrogen bonds in bottom panels are shown in yellow. Hydrogen atoms are not displayed.

To further explore the importance of this interfacial T:T pair to the structure and stability of the junction, we studied additional DNA constructs in which this T:T pair is either absent (**IDJ4**) or in a different relative orientation with respect the C:C^+^ stack (**IDJ5**). A junction with a C:C^+^ base-pair substituting the T:T mismatch was also explored (**IDJ6**) (Figures S14–S16). NMR and UV melting experiments show a behavior similar to the one observed in **IDJ1**. In all cases, the NMR spectra are consistent with formation of similar i-motif and duplex moieties. UV denaturation curves indicate lower stabilities for the i-motifs in **IDJ4** and **IDJ5**. In contrast, the i-motif in **IDJ6** is more stable (Table S1). Analyses of their NOESY spectra suggest that these junctions, in particular **IDJ5**, are more flexible than **IDJ1** at the site of the junction (Figures S17 and S18). The higher stability of **IDJ6** vs **IDJ4** and **IDJ5** is probably due to the presence of an additional C:C^+^ base-pair and the formation of an i-motif moiety with the 3′E topology. Interestingly, the orientation of the interfacial base-pair, T:T in **IDJ1** or C:C^+^ in **IDJ6**, may play an important role in the thermal stability of the IDJs.

The sequence in the hairpin moiety was a convenient choice for NMR studies, but other sequences, including biologically relevant ones, can form analogous junctions. For example, formation of an i-motif/hairpin junction has been proposed in the promoter region of the KRAS oncogene.^5a^ In vivo studies suggest that an i-motif-forming region of this promoter containing a stem/loop hairpin can be involved in regulation of KRAS expression by direct interaction with the transcription factor hnRNP K. NMR spectra of the native sequence strongly suggest that i-motif and duplex regions coexist at nearly neutral pH.^5a^ However, the quality of the NMR spectra was very poor. We explored the construct shown in Figure S20 (**IDJ7**), in which the part of the sequence farthest from the i-motif/duplex interface has been substituted by a sequence that forms a minor-groove tetrad without perturbing the i-motif/duplex interface. The resulting molecule exhibits well-dispersed NMR spectra, as shown in Figure S20, clearly showing the formation of an i-motif/duplex junction at neutral conditions.

In conclusion, i-DNA and B-DNA can coexist at physiological conditions, giving rise to i-motif/duplex interfaces (or junctions). Such interfaces can be conveniently studied by taking advantage of the stabilizing effect of minor-groove tetrads in i-motifs. The three-dimensional structure of the i/B-DNA junction determined here reveals that these two DNA structures are perfectly compatible, with no large disruptions in base-pair stacks. T:T base-pairs, which are known to be stabilizing capping elements in i-motifs, are also very well suited for stabilization of i-motif/duplex junctions. The distinctive groove shapes of this structure suggest that i/B-DNA junctions may be a motif recognized by proteins in the cell, which might be targeted by selective ligands.

## Supporting Information Available

Detailed descriptions of the experimental procedures and NMR assignments; Tables S1–S5 with assignments, calculation statistics, and structural analysis; Figures S1–S20 showing UV melting curves, CD, electrophoretic experiments, NMR data, and details on the structural models and sequence analysis (PDF)
