# Supplementary material for: Structure of i-Motif/Duplex Junctions at Neutral pH
Source: J Am Chem Soc. 2021 Aug 9;143(33):12919–23. doi: 10.1021/jacs.1c04679 (PMC8397320; doi:10.1021/jacs.1c04679)
Supplement: Supplementary file 1 — ja1c04679_si_001.pdf [file ja1c04679_si_001.pdf]

ELECTRONIC SUPPORTING INFORMATION

The structure of i-motif/duplex junctions at neutral pH.

Israel Serrano-Chacón[†], Bartomeu Mir[‡], Núria Escaja[‡,§]* and Carlos González[†,§]*.

[†]Instituto de Química Física 'Rocasolano', CSIC, Serrano 119, 28006 Madrid, Spain.

[‡] Inorganic and Organic Chemistry Department, Organic Chemistry Section, and IBUB, University of Barcelona, Martí i Franquès 1-11, 08028 Barcelona, Spain.

[§] BIOESTRAN associated unit UB-CSIC.

Corresponding Authors: nescaja@ub.edu; cgonzalez@iqfr.csic.es

Methods S-2

Supplementary Results S-3

Supplementary Figures

Figure S1: Melting experiments (NMR and UV) of **IDJ1**, **IDJ2** and **IDJ3** and proposed models. S-4
Figure S2: CD spectra of **IDJ1** at neutral pH and different temperatures. S-5
Figure S3: Native PAGE of several sequences forming IDJs. S-5
Figure S4: NMR spectra and UV melting curves of **IDJ1** at different oligonucleotide concentration. S-6
Figure S5: Non-exchangeable protons region of NOESY spectrum of **IDJ1**. S-7
Figure S6: ^{15}N-filtered ^{1}H NMR experiments for guanine imino assignments. S-8
Figure S7: Exchangeable protons region of NOESY spectrum of **IDJ1.** S-9
Figure S8: NMR spectra of **IDJ1-U10** and **IDJ1-U35** for resolving ambiguous thymine assignments. S-10
Figure S9: Regions of NOESY spectra of **IDJ1** and its ^{5m}C11 mutant showing key NOEs with T10-T35. S-11
Figure S10: Part of the H1'-H2'/H2" region of the DQF-COSY spectrum of **IDJ1**. S-12
Figure S11: Solution structure of **IDJ1** (ensembles). S-13
Figure S12. Base-pairs in the structure of **IDJ1**. S-14
Figure S13. Different views of the structure of **IDJ1** showing groove sizes. S-15
Figure S14: UV melting curves at neutral and acidic pH, NMR melting at pH 7 and scheme of **IDJ4.** S-16
Figure S15: UV melting curves at neutral and acidic pH, NMR melting at pH 7 and scheme of **IDJ5.** S-17
Figure S16: UV melting curves at neutral and acidic pH, NMR melting at pH 7 and scheme of **IDJ6.** S-18
Figure S17: Exchangeable protons region of NOESY spectrum of **IDJ4.** S-19
Figure S18: Exchangeable protons region of NOESY spectrum of **IDJ5.** S-20
Figure S19: Model of **IDJ4** and details of the interface. S-21
Figure S20: Rationale design of **IDJ7**. UV and NMR melting at pH 7. S-22

Supplementary Tables
Table S1: Oligonucleotide sequences studied in this report. S-23
Table S2: Chemical shift list of **IDJ1**, pH 7, T= 5°C. S-24
Table S3: Experimental constraints and calculation statistics of **IDJ1.** S-25
Table S4: Average dihedral angles and order parameters of the dimeric structure of **IDJ1.** S-26
Table S5. Average values of selected helical parameters of **IDJ1.** S-27

Supplementary References S-28

Methods

Oligonucleotides synthesis. Unlabelled oligodeoxynucleotides were purchased at IDT with standard desalting purification. Site-specific ^{15}N-labelled DNA oligonucleotides were chemically synthesized by β-cyanoethyl phosphoramidite method on an automated DNA synthesizer 3400 from Applied Biosystems using reagents from Glen Research and Cambridge Isotope Laboratories. ^{15}N-labelled phosphoramidite was diluted with unlabeled phosphoramidite to obtain an 5% ^{15}N-enrichment.[1] DMT-ON oligonucleotides were purified by Glen-Pak DNA Purifiction Cartridge. Samples were dissolved in Na^+ form.

CD and UV spectroscopy. Circular dichroism spectra were recorded on a Jasco J-815 spectropolarimeter. UV spectra were recorded on a Jasco V-730 spectrophotometer. Both fitted with a thermostated cell holder. Spectra were recorded in 25 mM sodium phosphate buffer at different pH values. Samples were initially heated at 90°C for 5 min and slowly allowed to cool to room temperature and stored at 4°C until use. UV melting curves were recorded at the wavelength of the larger positive band, ~ 265 nm, with a heating rate of 0.5 °C·min^{-1}. Associated error in T_m values determination has been estimated to be ± 0.2 °C.

Gel electrophoresis. Polyacrylamide gel electrophoresis under native conditions was performed to confirm the oligomeric state of the structures. 20% polyacrylamide gels (acrylamide/bisacrylamide 19:1) were prepared at pH 6 and supplemented with 0.05M phosphate buffer and 0.05M MES buffer. 20 µM oligonucleotide samples were prepared in buffer, renatured and maintained at 4 °C until use. Running buffer contained 0.05 M sodium phosphate buffer and 0.05M MES buffer at pH 6. Electrophoresis was performed at 200 V for 5 h. Gel visualization was achieved by using Stains-All (Sigma).

NMR. Samples for NMR experiments were dissolved (in Na^+ form) in either D_2O or 9:1 H_2O/D_2O, 25 mM sodium phosphate buffer. Most NMR experiments were carried out at pH 7, although some NOESY experiments were also performed at pH 6 to identify rapidly exchanging protons. The pH was adjusted by adding aliquots of concentrated solution of either HCl or NaOH. All NMR spectra were acquired on Bruker spectrometers operating at 600 and 800 MHz equipped with cryoprobes and processed with the TOPSPIN software. For the experiments in D_2O, presaturation was used to suppress the residual H_2O signal. A jump-and-return pulse sequence[2] was employed to observe the rapidly exchanging protons in 1D H_2O experiments. NOESY[3] spectra in D_2O and 9:1 H_2O/D_2O were acquired with mixing times of 100, 150 and 250 ms. TOCSY[4] spectra were recorded with the standard MLEV-17 spin-lock sequence and a mixing time of 80 ms. In most of the experiments in H_2O. water suppression was achieved by including a WATERGATE[5] module in the pulse sequence prior to acquisition. The spectral analysis program SPARKY was used for semiautomatic assignment of the NOESY cross-peaks and quantitative evaluation of the NOE intensities.

NMR constraints. Qualitative distance constraints were obtained from NOE intensities. NOEs were classified as strong, medium or weak, and distance constraints were set accordingly to 3, 4 or 5 Å. In addition to these experimentally derived constraints, hydrogen bond constrains for the base pairs were used. Target values for distances and angles related to hydrogen bonds were set to values obtained from crystallographic data in related structures.[6] Force constants were 20 kcal/mol.Å^2 for experimental distance constraints, and 30 kcal/mol.Å^2 for hydrogen bond distance constraints. Due to the relatively broad line-widths of the sugar proton signals, J-coupling constants were not accurately measured but roughly estimated from H1'-H2' and H1'-H2" DQF-COSY cross-peaks. When these cross-peaks were consistent with deoxyribose conformations in the general S-domain, sugar dihedral angles were constrained to the following target intervals: v0(-36.5°,-6.5°); v1(19.8°,49.8°); v2(-49.8°,-19.8°); v3(6.5°,36.5°) and v4(-15.0°,15.0°), with a force constant of 25 kcal/mol·rad. This is equivalent to loosely constrain the sugar pseudorotation phase angles (Ps) between 144° and 180°.

Structural calculations. Structures were calculated with the program CYANA 3.0[7] and further refined with the SANDER module of the molecular dynamics package AMBER 18.[8] Resulting CYANA structures were taken as starting points for the AMBER refinement, consisting of an annealing protocol in water, followed by trajectories of 500 ps each in which explicit solvent molecules were included and using the Particle Mesh Ewald method to evaluate long-range electrostatic interactions. The specific protocols for these calculations have been described elsewhere.[9] The BSC1 force field[10] was used to describe the DNA and the TIP3P model was used to simulate water molecules. Hemiprotonated $C:C^+$ base pairs were modelled by considering base pairs between neutral and protonated cytosine residues obtained from libraries included in the AMBER package. Analysis of the final structures was carried out with the program MOLMOL[11] and X3DNA.[12] We consider as representative structure the conformed with the lowest AMBER total energy. Coordinates are deposited in the PDB data bank (code 7O5E).

Supplementary Results

Sequence optimization. As a previous search for the optimal sequences for the structural determination of i-motif/duplex junctions by NMR methods, several oligonucleotides with sequences similar to **IDJ1** were studied (Figure S1). We explored: a) an oligonucleotide with a reduced number of C:C$^+$ base-pair in the i-motif part (**IDJ2**); and b) a construct in which formation of the minor groove G:T:G:T tetrad is impeded by swapping the guanines and thymine residues in positions 4 and 7 (**IDJ3**). As shown Figure S1, NMR spectra and UV melting experiments clearly show a significant destabilization in both cases. The lower stability of **IDJ2** reflects that the number of C:C$^+$ base-pair is a key factor in i-DNA stability, as noticed in previous studies.[13] More interesting is the case of **IDJ3**, in which a minimal change in the sequence hampering the G:T:G:T tetrad formation provokes a dramatic decrease in the melting temperature of the i-motif part (13 °C). The NMR spectra of **IDJ3** with only two signals in the 15-16 ppm region show that many of the C:C$^+$ base pairs displayed in the presumed model are not formed at neutral pH. These results highlight the importance of capping minor groove tetrads for i-motif stabilization.

Since a balance must be struck between thermal stability and a reasonable molecular size for NMR studies, we decided to focus on **IDJ1** for a detailed structural characterization of an i-motif/duplex junction.

Assignment of the NMR spectra. The starting point of the assignment was the imino signals of Watson-Crick A:T base pairs. The imino resonance at 13.99 ppm was assigned to T21 by its cross-peak with a guanine imino signal at 12.84 ppm that was assigned to G14 (the only G:C - A:T step in the sequence). Then, C22 amino signals were assigned by their cross-peaks with H1G14. The whole C22 spin system was identified by amino→H5→H6→sugar connectivity. After this, all the residues in the duplex moiety were identified by their sequential sugar-aromatic NOEs, following standard procedures (Figure S5).

NOE cross-peaks between imino protons in the 10-12 ppm region indicate the formation three G:T or T:T base pairs. Guanine imino signals of the guanines 4, 25, and 29 were identified by performing ^{15}N-filtered experiments in samples 5% enriched with ^{15}N-labelled guanines (see Figure S6). The corresponding base-paired thymines (T7 and T32) were assigned by their H1G-H3T cross-peaks (see Figure S7). The remaining imino-imino cross-peak corresponds to the T10:T35 base pair. A number of NOEs between protons of G:T:G:T tetrad with hemiprotonated cytosine protons allowed the assignment of C3 and C28. Their H2' protons exhibit a characteristic chemical shift (1.1 ppm). The rest of the cytosines in the i-motif were assigned following the particular sugar-sugar cross-peaks through the i-motif minor groove, and H5-H5 through the major groove. Several NOEs between T10:T35 with C1 and C26 in the i-motif and C11:G26 in the duplex moiety were essential to confirm the complete assignment. Although the thymine spin systems involved in the T:T base pair were clearly identified, the specific assignment of imino protons to either T10 or T35 was ambiguous. To resolve this uncertainty, oligonucleotides incorporating 2'-deoxyuridine (dU) substitutions at either position 10 or 35 were studied. The imino and methyl regions of their NMR spectra are shown in Figure S8. Chemical shifts changes with respect to **IDJ1** allowed the specific assignment of T10 and T35. These assignments were further checked by analyzing the NMR spectra of an oligonucleotide incorporating a 5-methylcytosine (^{5m}C) residue at position 11. Detection of key cross-peaks with 5-methyl of ^{5m}C11, and the comparison with the unmodified **IDJ1** construct (Figure S9) confirmed the complete unambiguous assignment of T10 and T35.

Supplementary Figures

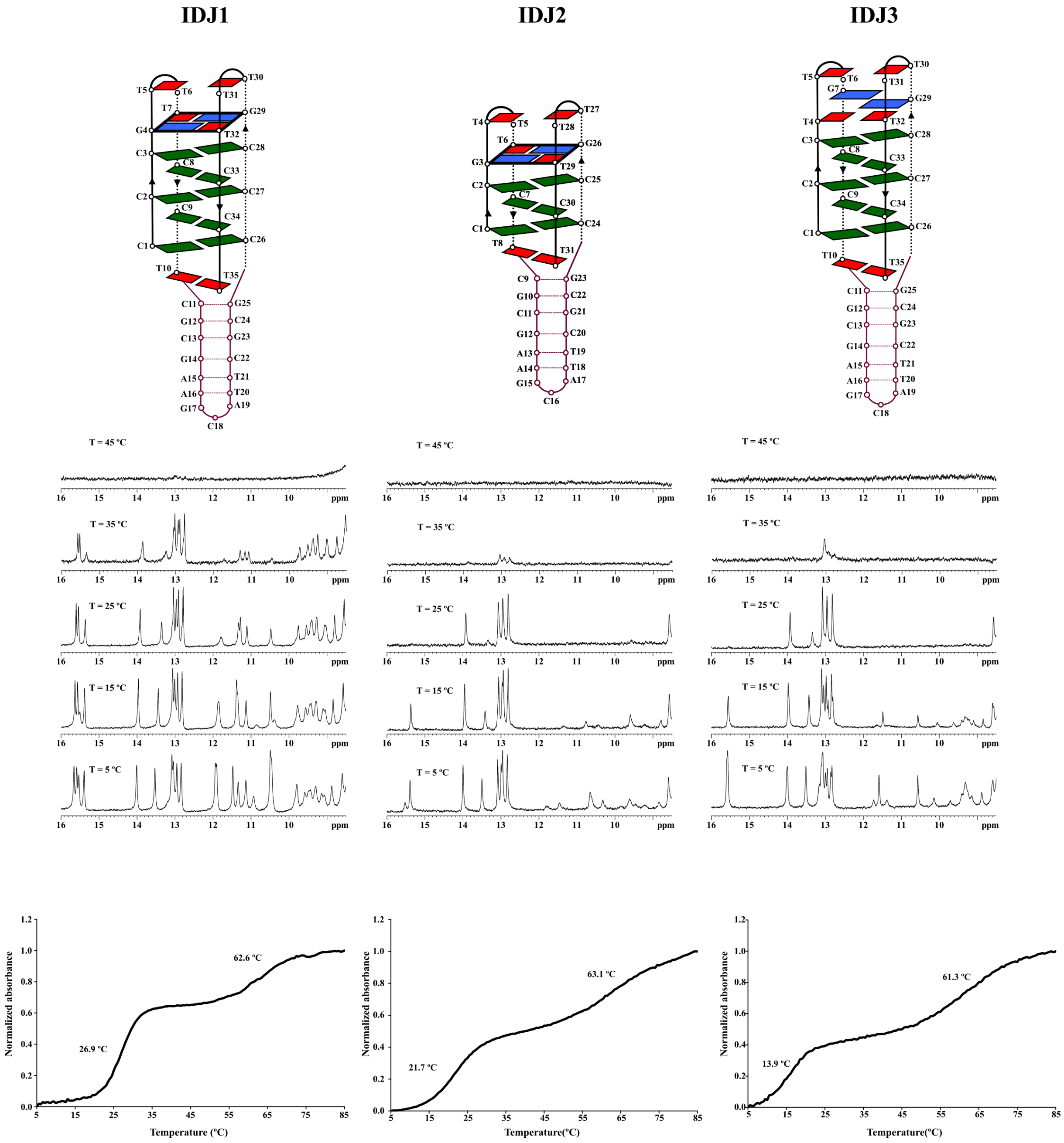

Figure S1. Top) Proposed structural models of **IDJ1**, **IDJ2** and **IDJ3.** Middle) Exchangeable proton regions of the NMR spectra at different temperatures. Bottom) UV melting experiments. Buffer conditions: 25 mM sodium phosphate, pH 7. [oligonucleotide]=0.5 mM for NMR, and 2 μM for UV experiments.

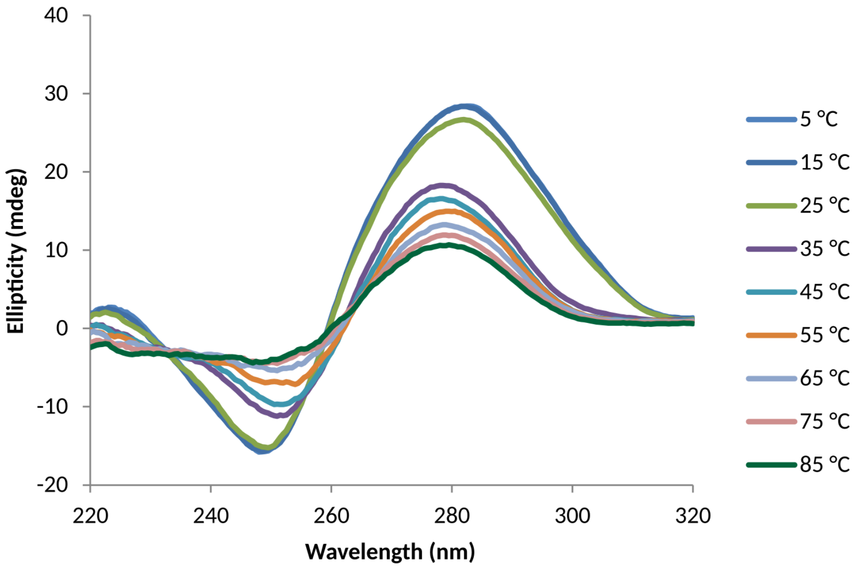

Figure S2. CD spectra of **IDJ1** at different temperatures at pH 7 (10 mM sodium phosphate buffer, [oligonucleotide]=20 µM).

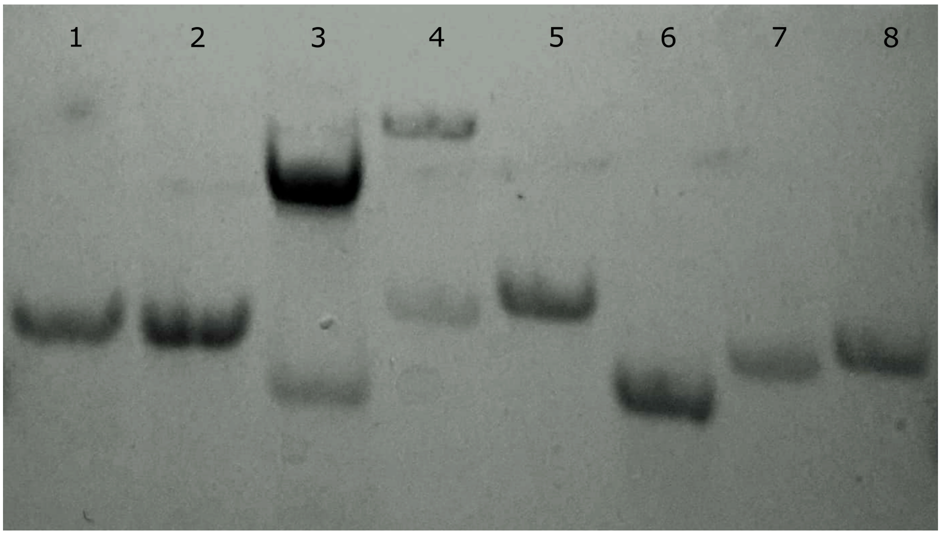

Figure S3. Native PAGE of several DNA sequences containing i-motif/duplex junctions and their controls. Lane 1: **IDJ5** (35mer junction); lane 2: **IDJ4** (33 mer junction); lane 3: control duplex (self-complementary 26mer); lane 4: **LL4-DDD** hybrid (35mer sequence consisting of **LL4** mini i-motif attached to the Dickerson-Drew dodecamer sequence, **DDD**); lane 5: **LL4-hairpin** hybrid (38mer sequence consisting of **LL4** mini i-motif attached to the same stem/loop hairpin as **IDJ1**); lane 6: **IDJ2** (31mer junction); lane 7: **IDJ1** (35mer junction); lane 8: **IDJ3** (35mer junction).

Control duplex and **LL4-DDD** contain self-complementary sequences and, therefore, they can form dimeric structures of 52 and 70mer respectively, as can be clearly seen in lanes 3 and 4. All other oligonucleotides adopt monomeric structures in the gel.

Control duplex: 5’ -dCAATCGGATCGAATTCGATCCGATTG -3’
LL4-DDD: 5’-dTCGTTCCGTTTTTTCGTTCCGT-T-CGCGAATTCGCG -3
LL4-hairpin: 5’-dTCGTTCCGTTTTTTCGTTCCGT-T- CGCGAAGCATTCGCG-3’

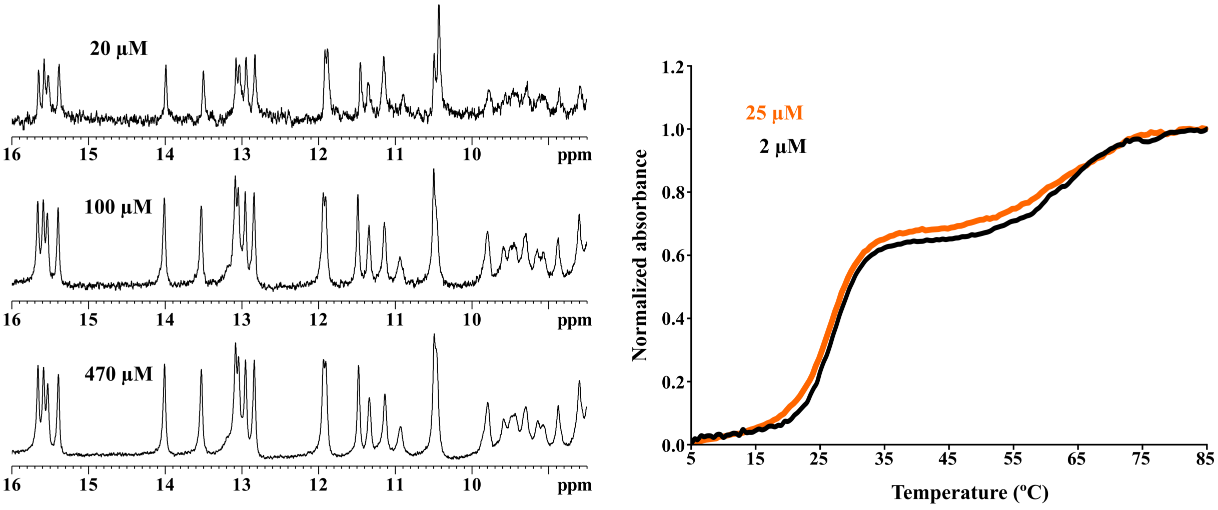

Figure S4. Left) NMR spectra of **IDJ1** at different oligonucleotide concentrations (T=5 °C). Right) UV melting curves of **IDJ1** at two different oligonucleotide concentrations. T_m values for the i-motif denaturation were 26.9 °C and 26.1 °C at 2 μM and 25 μM DNA concentration, respectively. Buffer conditions: 25 mM sodium phosphate buffer, pH 7.

T_m values for denaturation curves are independent of oligonucleotide concentration within the experimental error, confirming that the structure of **IDJ1** is monomeric at low DNA concentration, in agreement with native PAGE results. Since the NMR spectra do not change at DNA concentrations ranging from 20 μM to 500 μM, we can conclude that the structure is monomeric at the adequate conditions for recording 2D spectra.

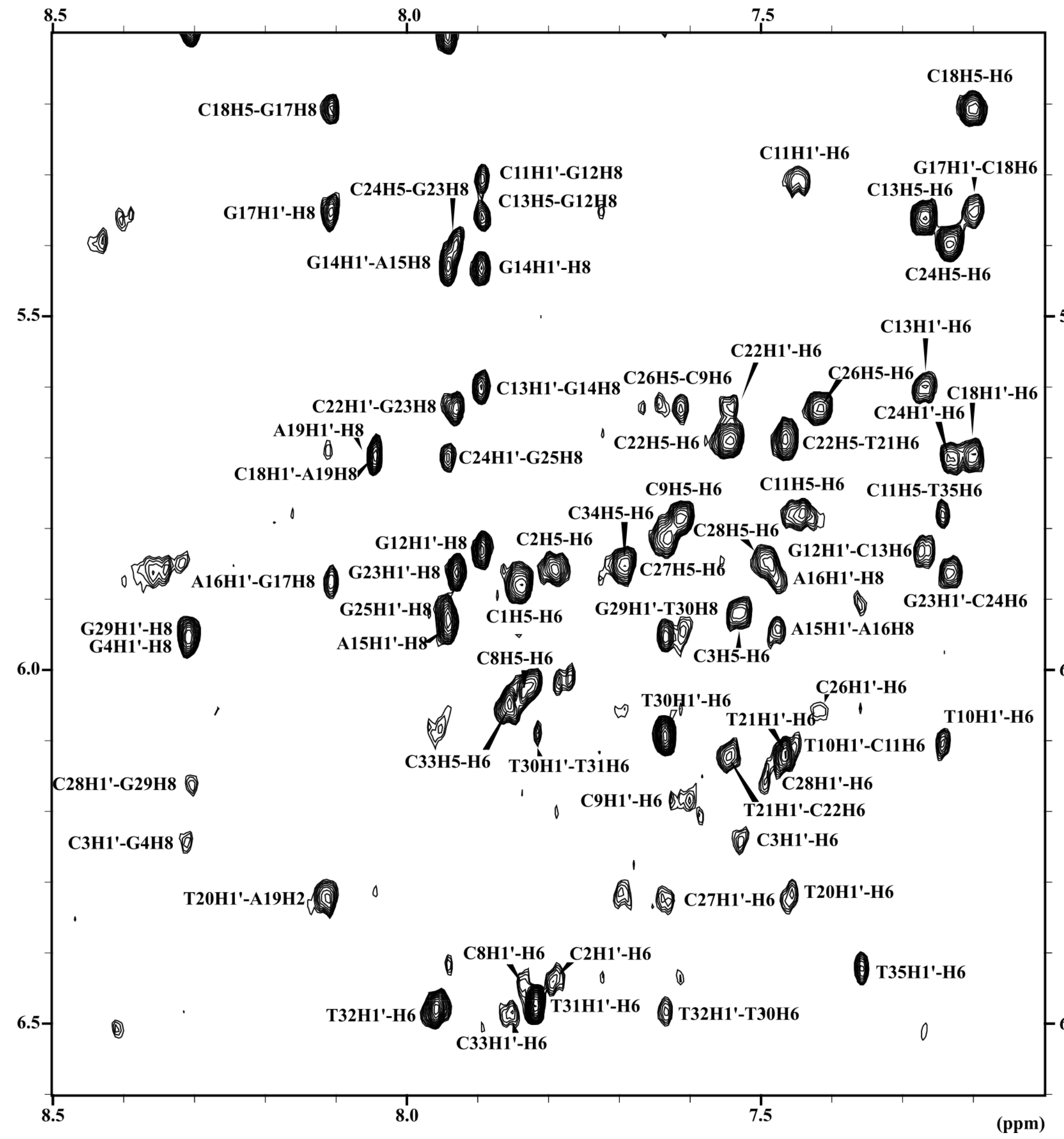

Figure S5. Non-exchangeable protons regions (aromatic-H5/H1') of NOESY spectrum (250 ms mixing time) of **IDJ1**, 25 mM sodium phosphate buffer, pH 7, T=5°C, [oligonucleotide]=0.5 mM.

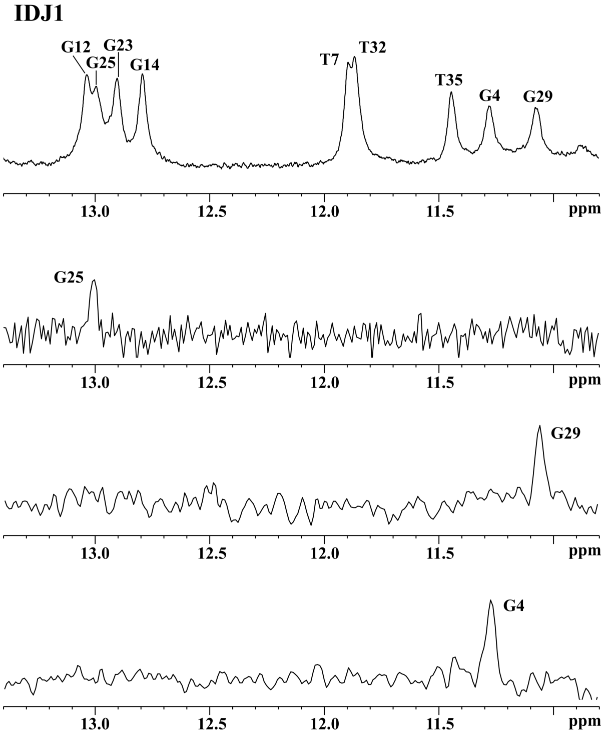

Figure S6. Assignment of some guanine imino protons of **IDJ1** with the help of site-specific 5% ^{15}N-labelled guanines (25 mM sodium phosphate buffer, pH 7, T=5 °C, [oligonucleotide]=0.5 mM). Other guanine imino protons in the hairpin were unambiguously assigned through standard ^{1}H NMR methods.

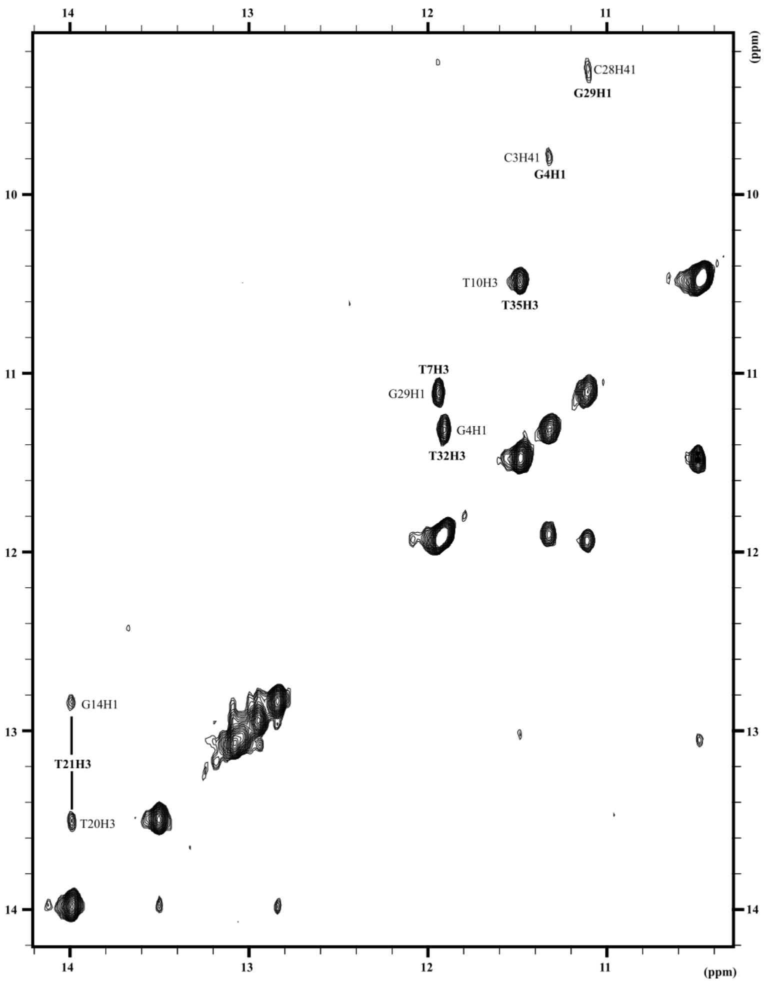

Figure S7. Exchangeable protons regions of NOESY spectrum (mixing time 150 ms) of **IDJ1**. 25 mM sodium phosphate buffer, pH 7, T=5°C, [oligonucleotide]=0.5 mM. Of particular interest are the imino-imino NOEs supporting the T10:T35 base pair, and G4:T32 and G29:T7 base pairs involved in the tetrad.

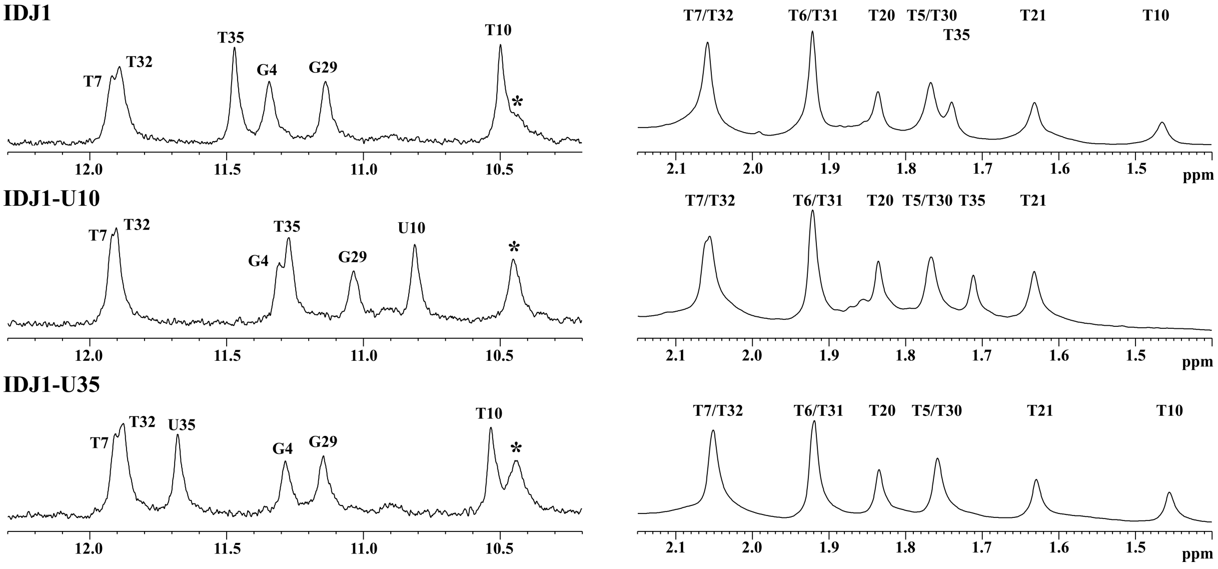

Figure S8. ^{1}H-NMR spectra of **IDJ1** and the T-> dU mutants **IDJ1-U10** and **IDJ1-U35** (25 mM sodium phosphate buffer, pH 7, T=5 °C, [oligonucleotide]= 0.5 mM). The imino protons region is shown on the left and the methyl region on the right. Methyls of T10 and T35 can be directly assigned by checking the missing signals in **IDJ1-U10** and **IDJ1-U35** spectra. Imino signal of T10 could be assigned by comparing the spectra of IDJ1-U35 and IDJ1, and confirmed by the changes in **IDJ1-U10** spectra. "*" indicates and unassigned imino signal, most probably belonging to T5/T30. Lack of cross-peaks in the NOESY spectra impedes its assignment.

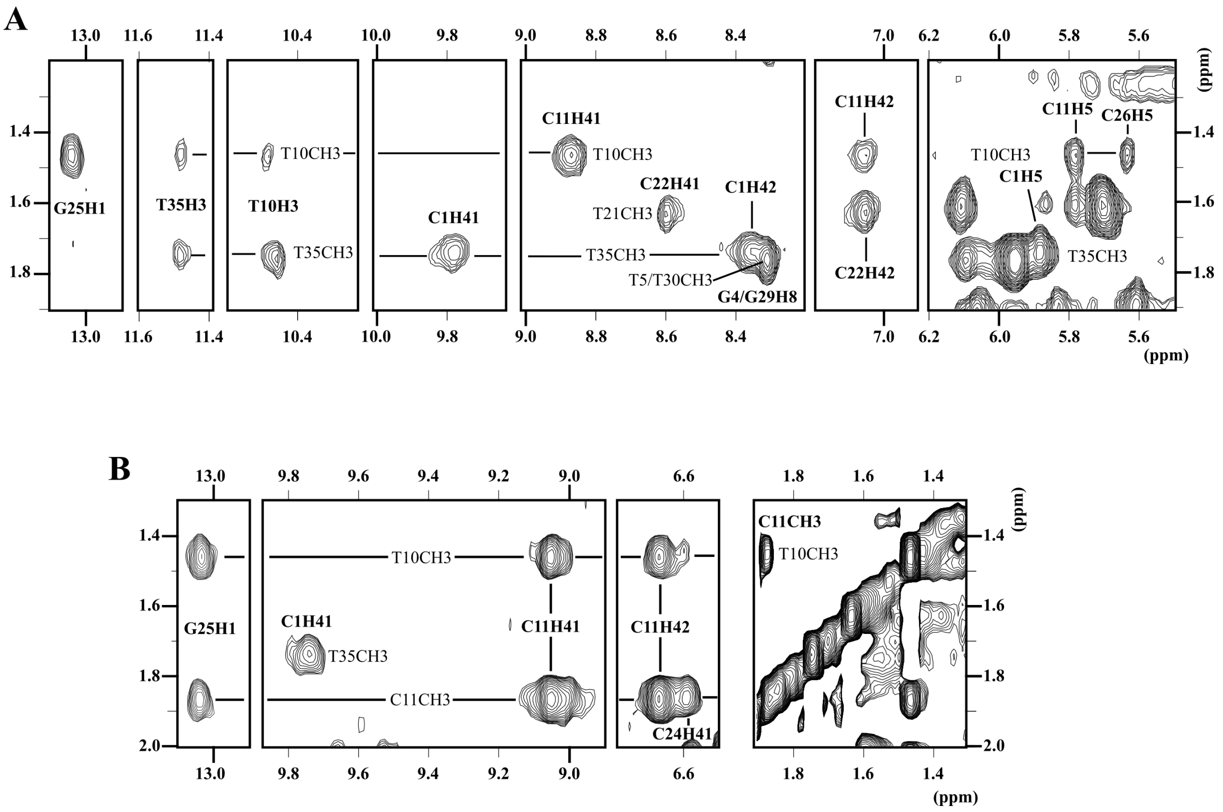

Figure S9. Several regions of the NOESY spectra (150 ms) of **IDJ1** (top) and the C-> ^{5m}C mutant **IDJ1-^{5m}C11** (bottom) (25 mM sodium phosphate buffer, pH 7, T=5 °C, [oligonucleotide]= 0.5 mM). Key NOEs connecting T10 and T35 with surrounding residues at the i-motif/duplex interface are shown. Of particular interest are the NOEs supporting the T10:T35 base pair at the i-motif/duplex interface, such as T10H3-T35CH3 and T35H3-T10CH3.

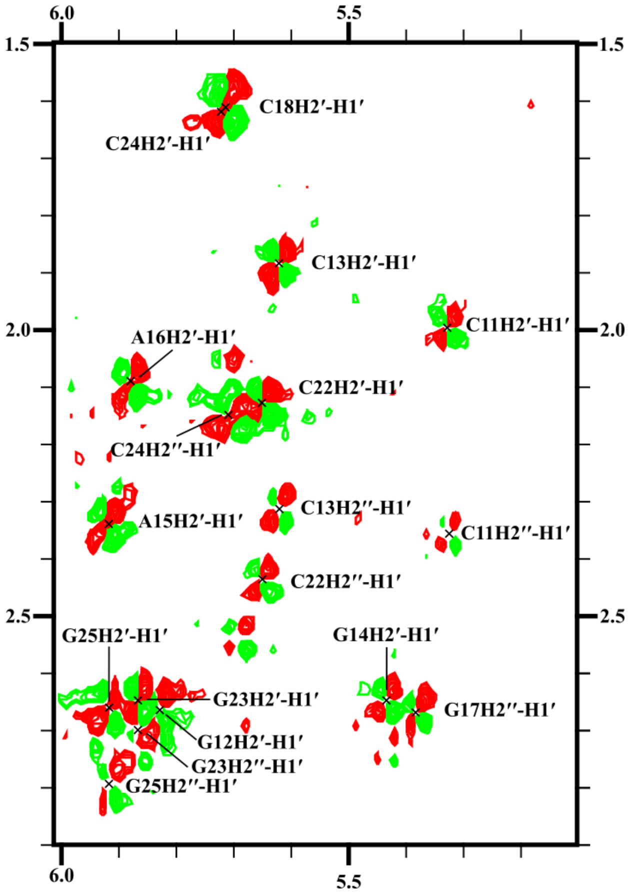

Figure S10. Region of H1'-H2'/2" region of a DQF-COSY spectrum of **IDJ1** (25 mM sodium phosphate buffer, pH 7, T=20°C, [oligonucleotide]=0.5 mM). In spite of the large signal linewidths, some nucleotides exhibit $^3J_{1'2'}$ clearly larger than $^3J_{1'2''}$. indicating that their sugar conformation is mainly in the South domain. Dihedral angles for residues C3, C13, G14, A15, A16, T20, T21, C22, G23 and C28 were restrained to the South domain as explained in the Methods Section.

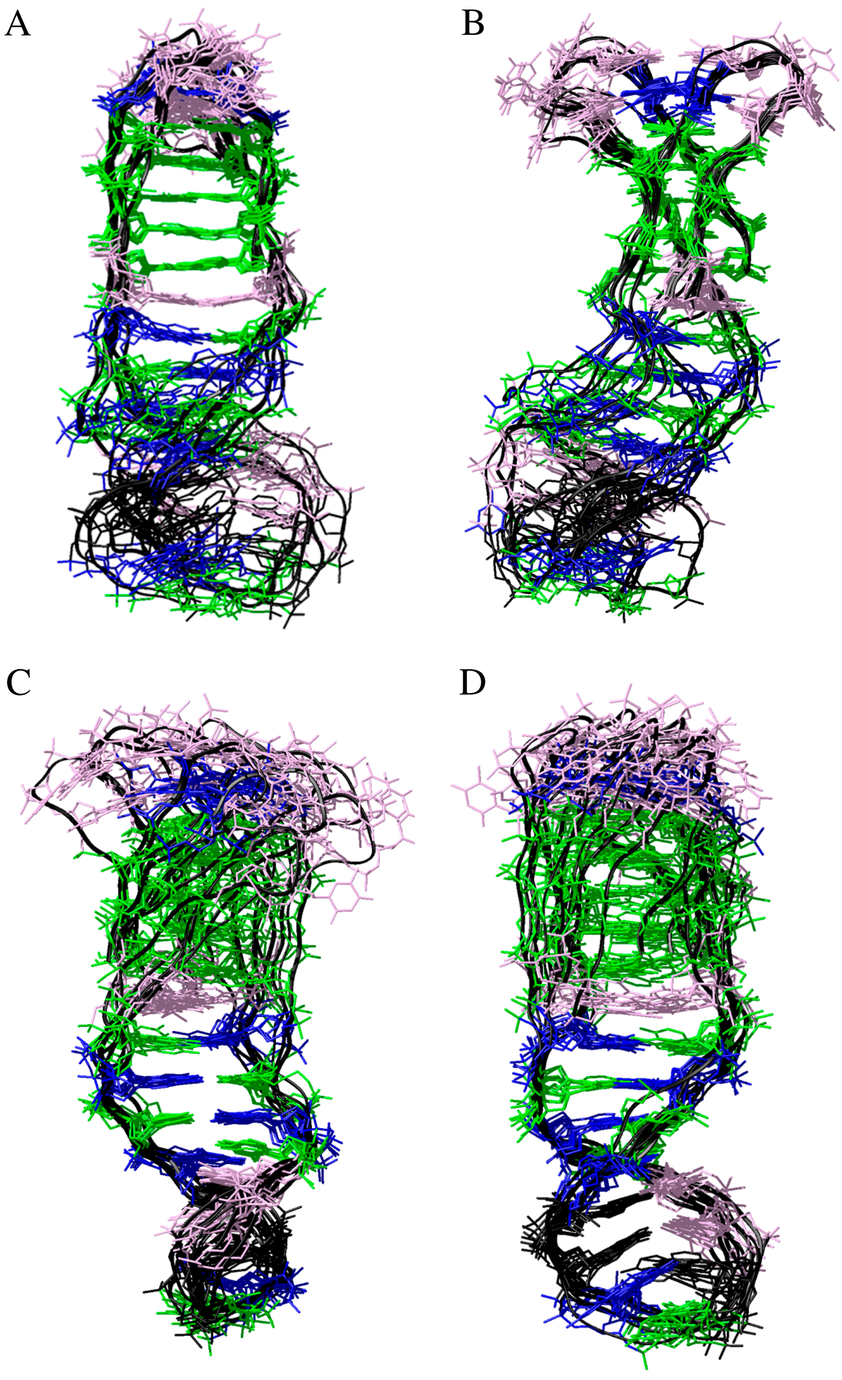

Figure S11. Several views of the ensemble of the ten resulting structures of **IDJ1**. Top: Structural superposition considering the heavy atoms in the i-motif moiety. Views from the major (A) and the minor groove (B) of the i-motif. Bottom: Structural superposition considering the heavy atoms in the stem (duplex) moiety. Views from the major (C) and the minor groove (D) of the duplex. Colour code: Cytosines in green, guanines in blue, thymines in pink, and adenines in black. PDB code 7O5E.

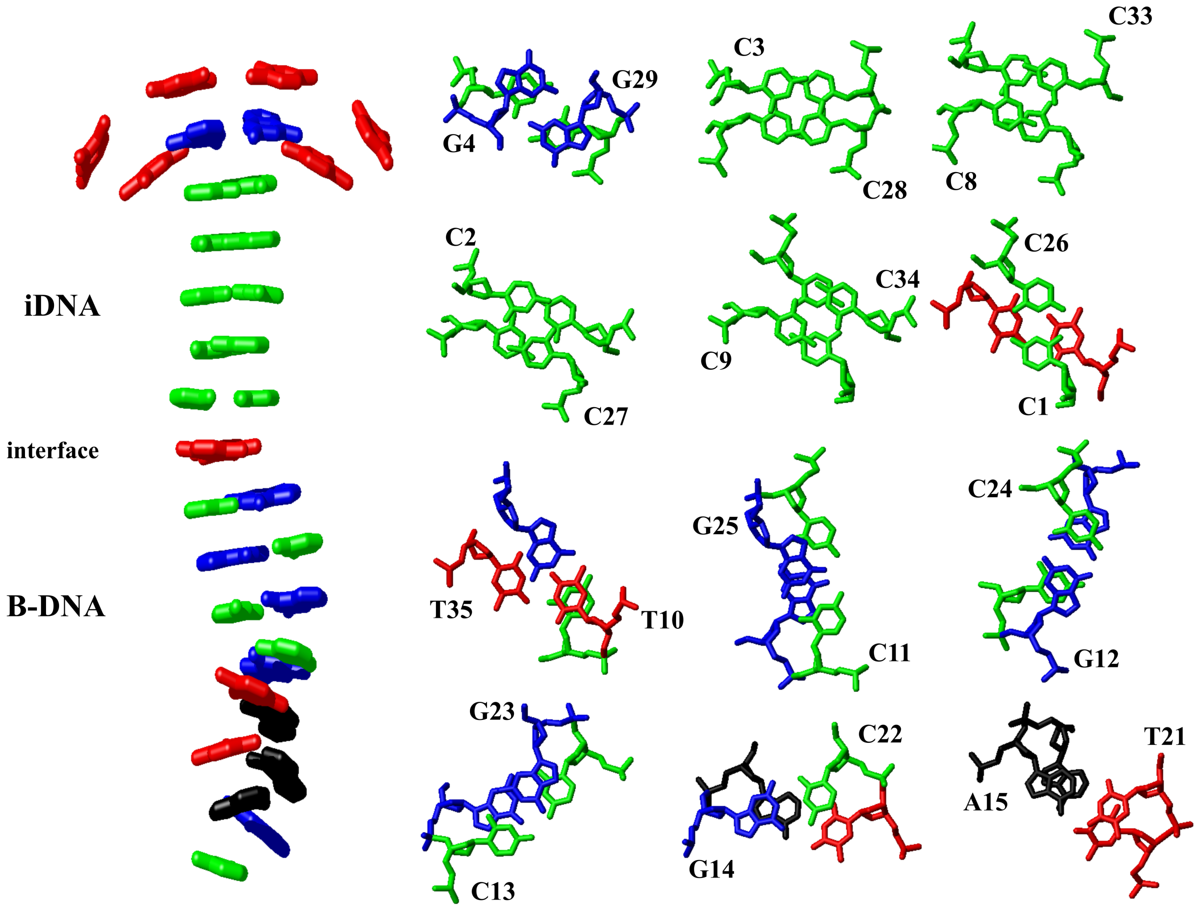

Figure S12. Base-pairs in the structure of **IDJ1**. Left) the complete base-pairs stack. Right) Details of the stacking interactions in all the base-pairs steps. Colour code: Cytosines in green, guanines in blue, thymines in red and adenines in black.

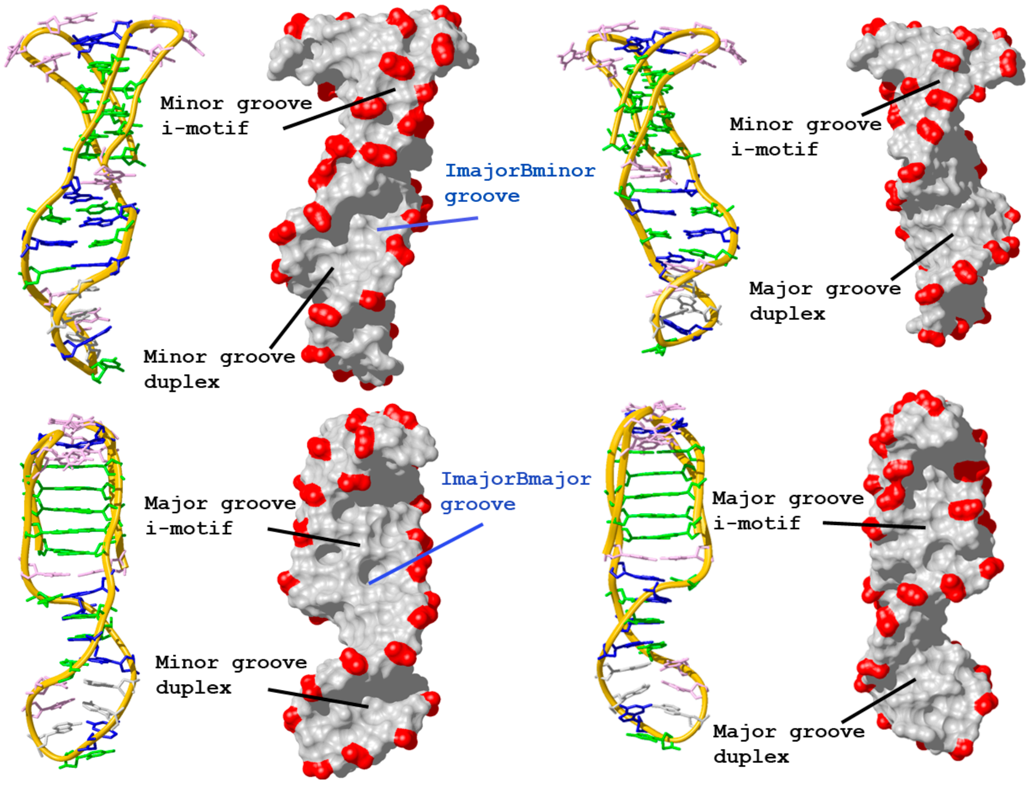

Figure S13. Several views of **IDJ1** structure. The different grooves are indicated. The i-motif region forms the four characteristic grooves: two of them very broad (major grooves), and the other two extremely narrow (minor grooves). In this particular structure the two i-motif major grooves present different widths (bottom panels). Each of these grooves connects with the major and minor grooves of the duplex region, giving rise to two overall grooves, named as ImajorBmajor and ImajorBminor respectively.

Colour code: Cytosines in green, guanines in blue, thymines in pink, and adenines in grey. Phosphates are shown in red in the surface representations.

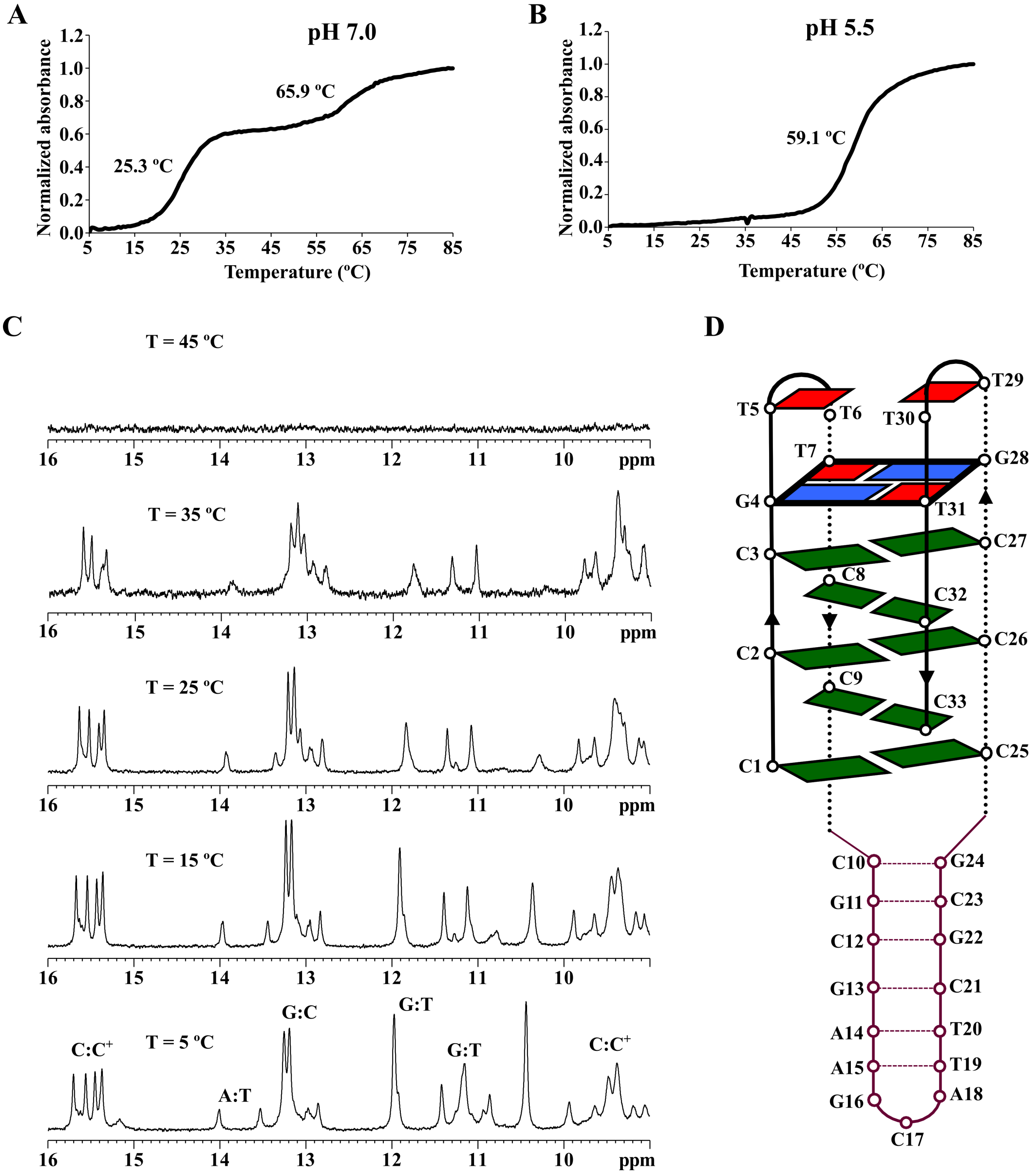

Figure S14. UV melting curves of **IDJ4** at neutral (A) and acidic pH (B), [oligonucleotide]=2 µM . Exchangeable proton spectra of **IDJ4** at different temperatures, pH 7, [oligonucleotide]=0.5 mM (C). Buffer: 25 mM sodium phosphate. Scheme of the structure of **IDJ4**. Colour code: cytosines in green, guanines in blue, and thymines in red (D).

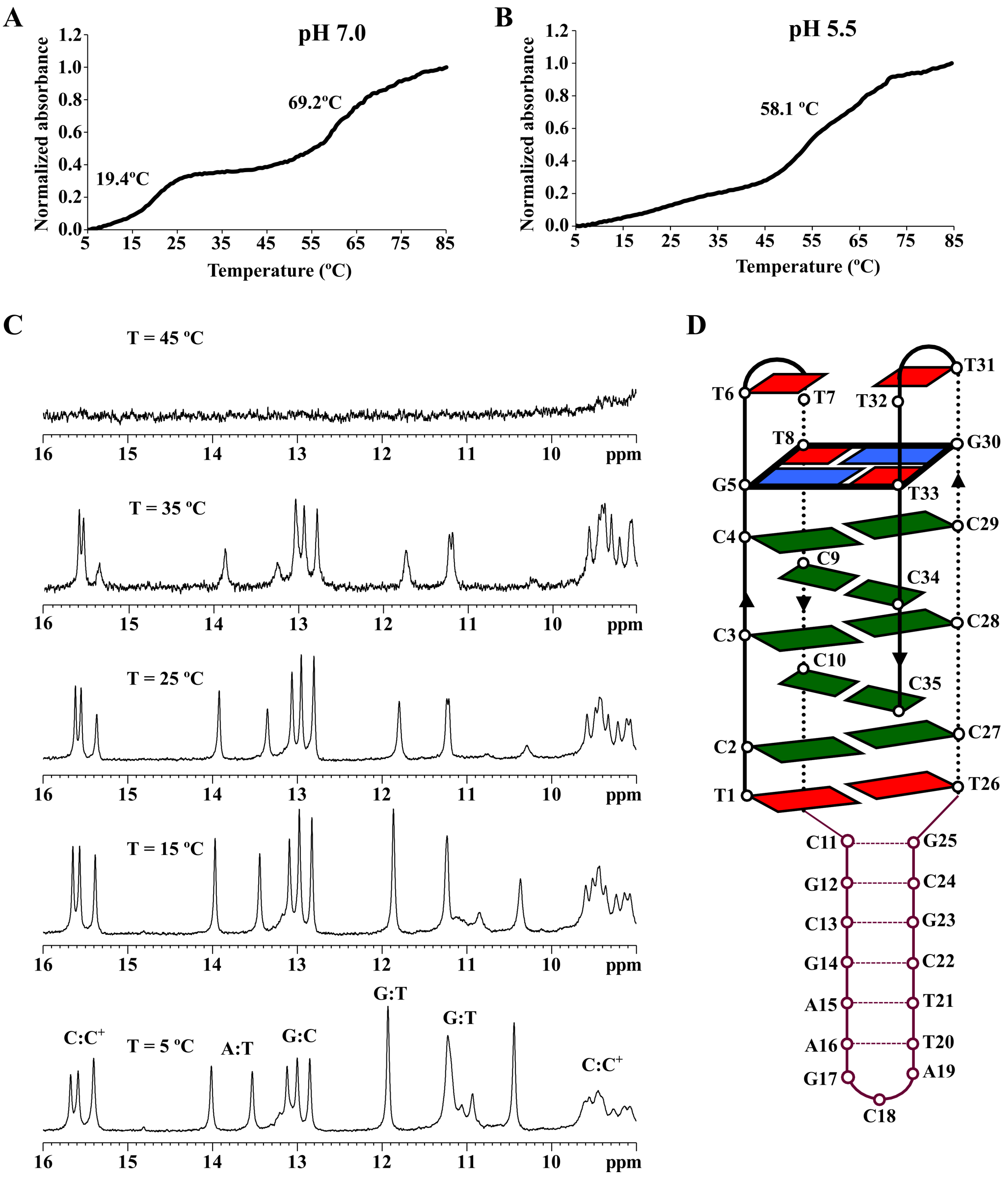

Figure S15. UV melting curves of **IDJ5** at neutral (A) and acidic pH (B), [oligonucleotide]=2 µM . Exchangeable proton spectra of **IDJ5** at different temperatures, pH 7, [oligonucleotide]=0.5 mM (C). Buffer: 25 mM sodium phosphate. Scheme of the structure of **IDJ5**. Colour code: cytosines in green, guanines in blue, and thymines in red (D).

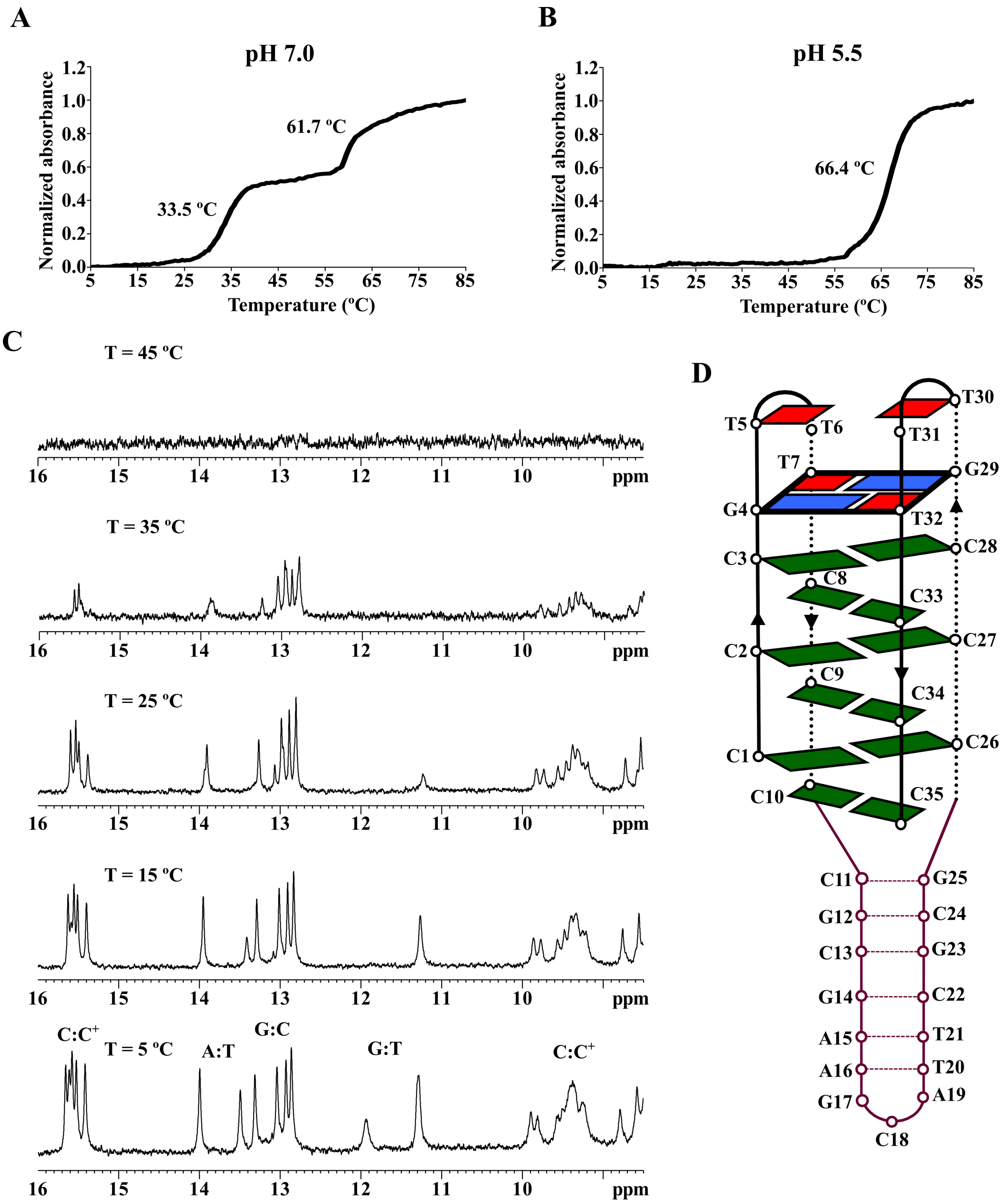

Figure S16. UV melting curves of **IDJ6** at neutral (A) and acidic pH, [oligonucleotide]=2 µM (B). Exchangeable proton spectra of **IDJ6** at different temperatures, pH 7, [oligonucleotide]=0.5 mM (C). Buffer: 25 mM sodium phosphate). Scheme of the structure of **IDJ6**. Colour code: cytosines in green, guanines in blue, and thymines in red (D).

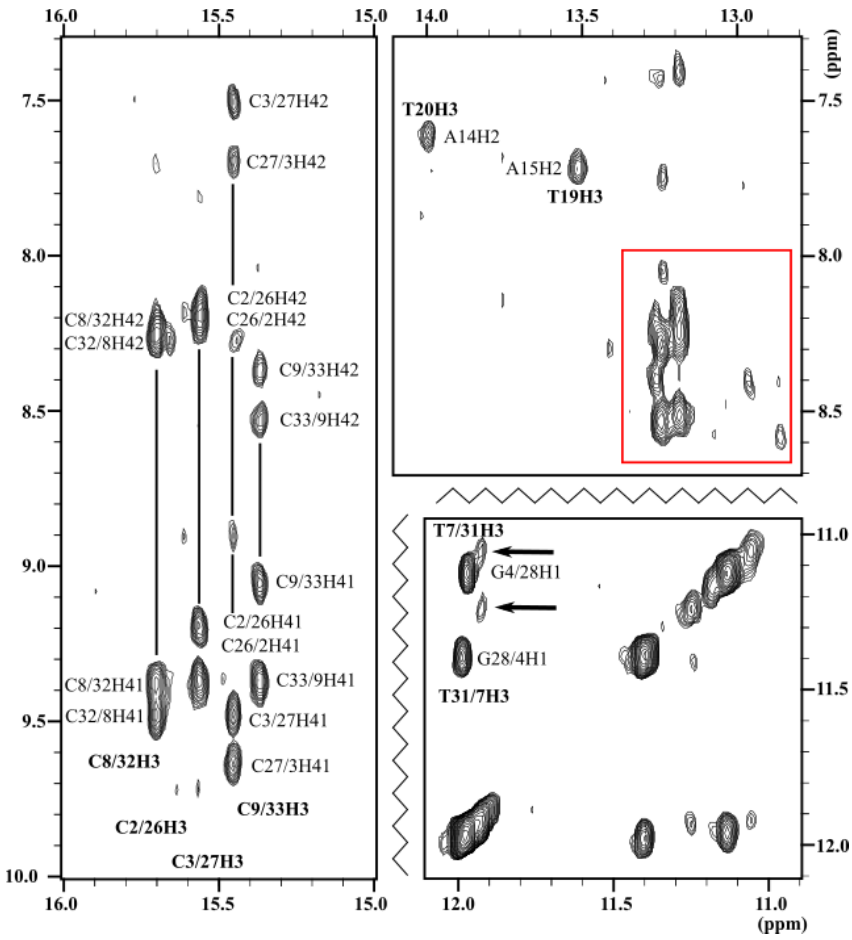

Figure S17. Exchangeable protons regions of NOESY spectrum (mixing time 150 ms) of **IDJ4**. 25 mM sodium phosphate buffer pH 6, T=5 °C, [oligonucleotide]=0.5 mM. Four cytosine imino signals with their imino-amino cross-peaks corresponding to C:C⁺ base pairs are observed (left panel). Two G:T base pairs were detected by their H1G-H3T and other cross-peaks (right bottom panel). Assignment of these resonances could be achieved by following the same procedures as in **IDJ1**. Watson-Crick base pairs corresponding to the stem regions are also observed (right up panel). Six imino proton resonances are observed in the Watson-Crick region (panel top right and Figure S14C). indicating that all base-pairs in the stem are formed. A:T base pairs could be assigned, but the presence of some minor species prevented the unambiguous assignment of all G:C base pairs (red square right upped panel). Arrows indicate cross-peaks from a minor species.

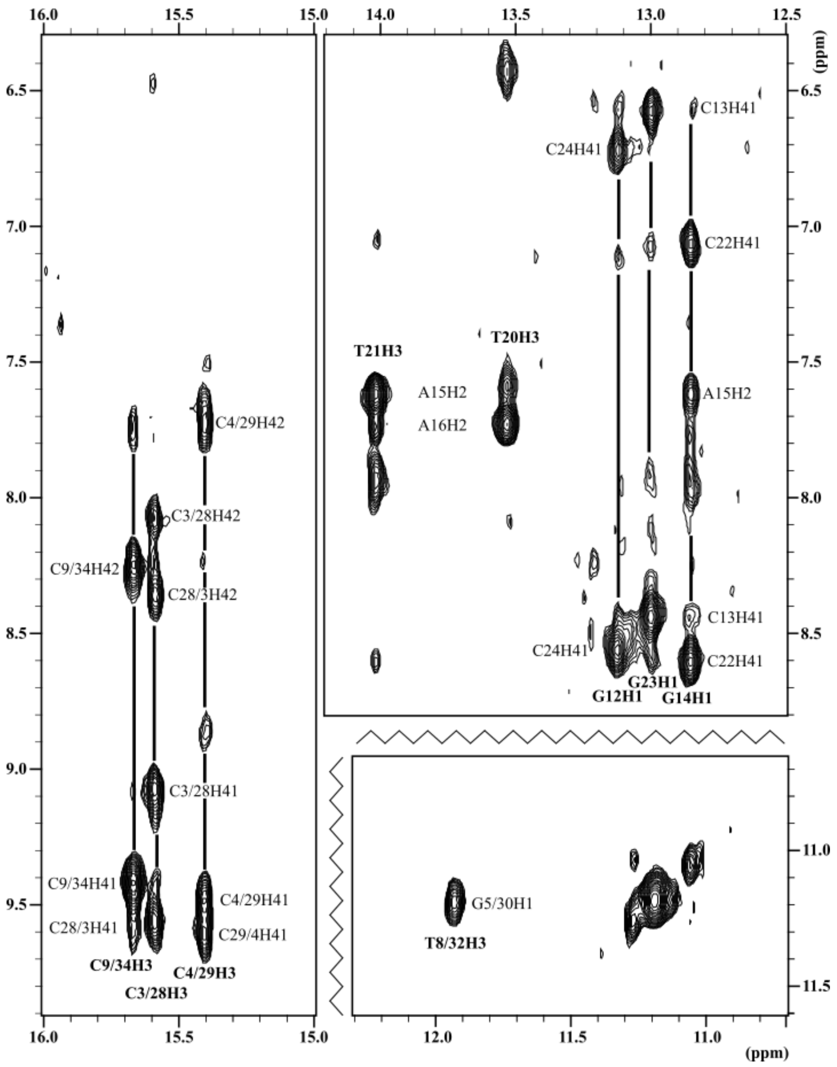

Figure S18. Exchangeable protons regions of NOESY spectrum (mixing time 150 ms) of **IDJ5**, 25 mM sodium phosphate buffer pH 6, T=5 °C, [oligonucleotide]=0.5 mM. In this case, only three cytosine imino signals with their imino-amino cross-peaks corresponding to C:C$^+$ base pairs are observed (left panel). G:T base pairs were detected by their H1G-H3T and other cross-peaks. Watson-Crick base pairs corresponding to the stem regions are also observed (right panel). Two A:T and three C:G base pairs (top right) were assigned by following the same procedure as in the case of **IDJ1**. No signals corresponding to the T:T base pairs at the interface could be detected.

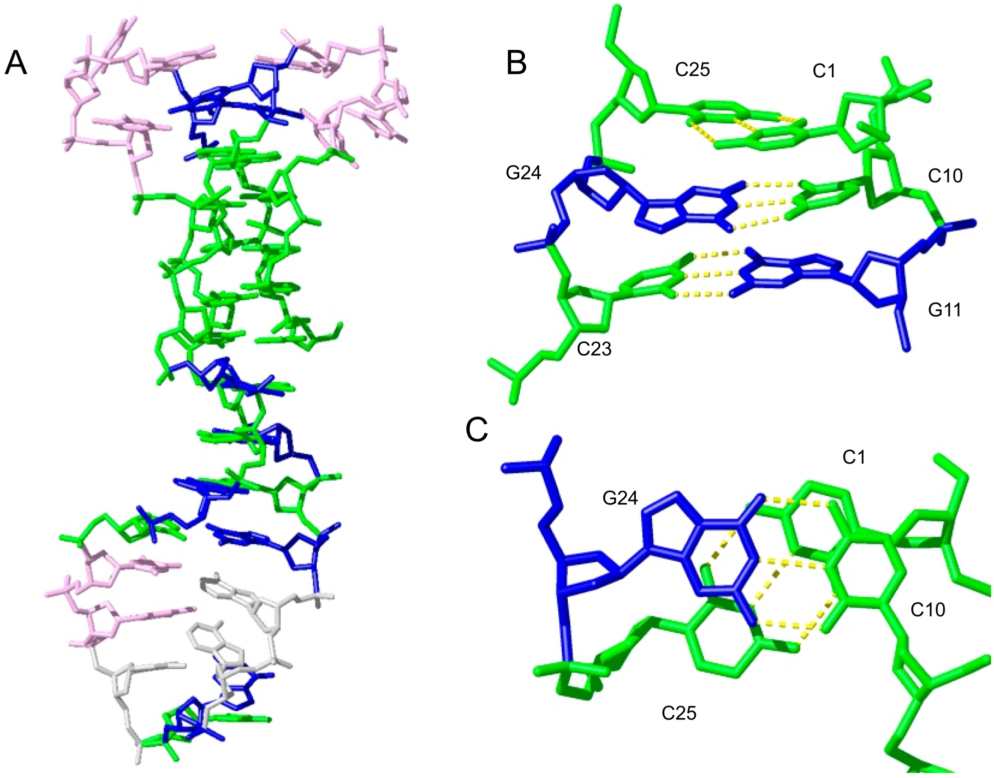

Figure S19: Computational model of **IDJ4** based on NMR data. A) Overall view. B) Lateral view of the junction showing the three base-pairs involved in the interface: C1:C25⁺, G24:C10 and C23:G11. C) Detail of the interface showing the stacking between the interfacial C1:C25⁺ base pair and the Watson-Crick G24:C10 base pair. Colour code: Cytosines in green, guanines in blue, thymines in pink and adenines in grey.

This model structure was obtained on the basis of **IDJ1** structure. After performing the appropriated sequence deletions and mutations, the structure was submitted to 10 ps of unrestricted molecular dynamics calculation following the same protocols used for the structural determination of **IDJ1**. Although NMR signals at the i-motif/duplex interface could not be assigned, the resulting model is consistent with the number of exchangeable proton signals observed in the spectra and suggests a direct stacking interaction between the interfacial hemiprotonated C1:C25⁺ base pair and the Watson-Crick G24:C10 base pair.

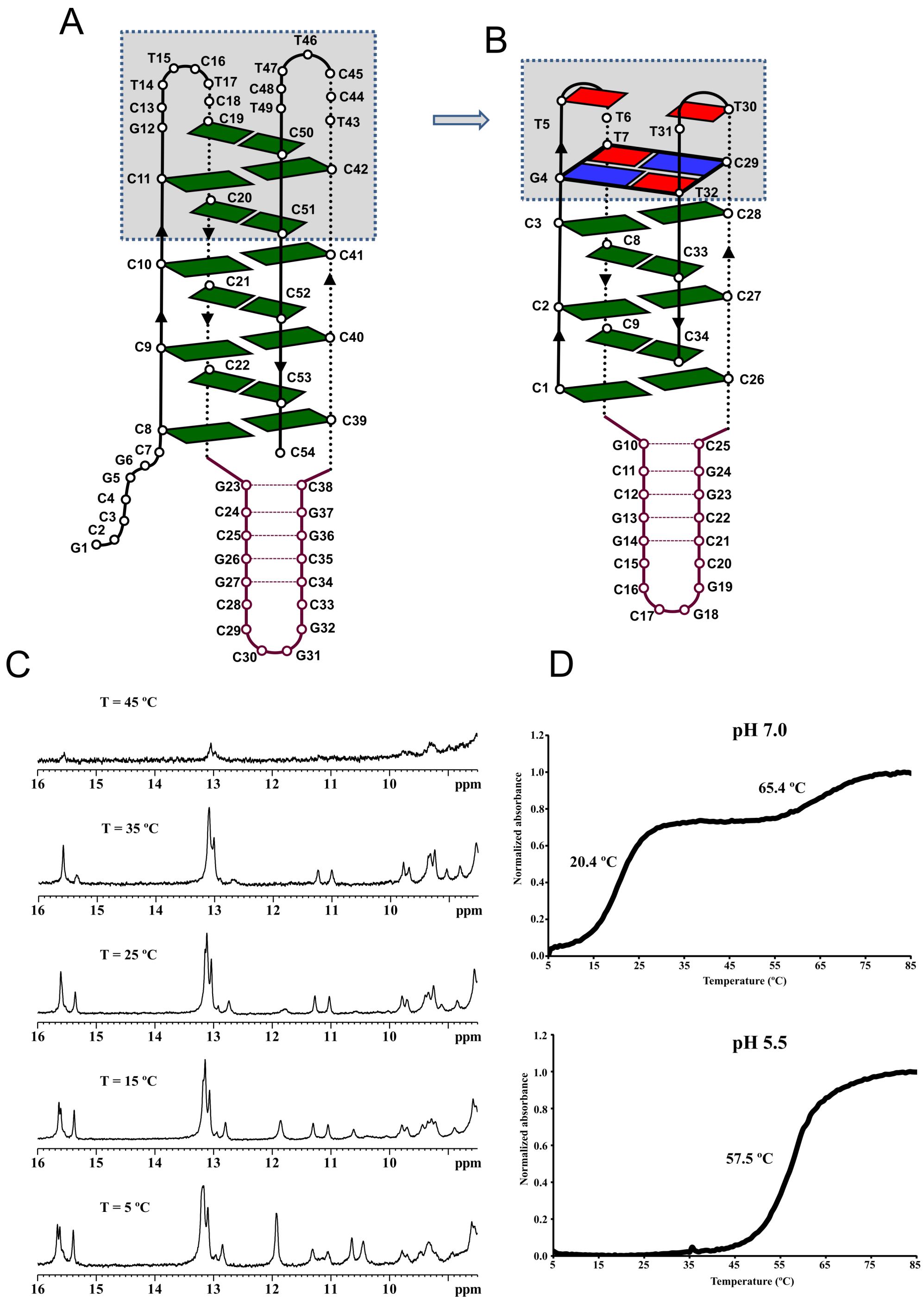

Figure S20: Rationale for **IDJ7** design. A) Illustration of the i-motif structure proposed for the cytosine-rich regions in the KRAS oncogene promoter. B) DNA construc proposed as potential analogue of the interface region (**IDJ7**). Modifications affect a region very distant from the interface between the i-motif and the duplex regions. The proposed modification consists of substituting the region shown in grey squares by a minor groove G:T:G:T tetrad with shorter loops.

NMR spectra of the native sequence suggest the simultaneous formation of i-motif and duplex structures at nearly neutral pH (Kaiser et al. Figure 3E), but NMR signals are very broad, impeding any further analysis. (C) Exchangeable proton spectra of **IDJ7** at different temperatures, showing narrow and well-dispersed signals (25 mM sodium phosphate buffer, pH 7, [oligonucleotide]=0.5 mM). D) UV melting curves of **IDJ7** at neutral and acidic pH, [oligonucleotide]= 3 µM.

Supplementary Tables

Table S1. Oligonucleotide sequences studied in this paper, and melting temperatures monitored by UV at 25 mM sodium phosphate buffer, pH 7.

Name	Sequence#	T_m (ºC)*
IDJ1	5'-CCCGTTTCCTCGCGAAGCATTCGCGCCCGTTTCCT-3'	26.9/62.6
IDJ2	5'-CCGTTTCTCGCGAAGCATTCGCGCCGTTTCT-3'	21.7/63.1
IDJ3	5'-CCCTTTGCCTCGCGAAGCATTCGCGCCCGTTTCCT-3'	13.9/61.3
IDJ4	5'-CCCGTTTCCCGCGAAGCATTCGCGCCCGTTTCC-3'	25.3/65.9
IDJ5	5'-TCCCGTTTCCCGCGAAGCATTCGCGTCCCGTTTCC-3'	19.4/69.2
IDJ6	5'-TCCCGTTTCCCGCGAAGCATTCGCGCCCCGTTTCC-3'	33.5/61.8
IDJ7	5'-CCCGTTTCCGCCGGCCCGGCCCGGCCCCGTTTCC-3'	20.4/65.4

# Nucleotides forming the i-motif are shown in black, and those in the hairpin moiety in cyan.

* First transitions correspond with i-motif denaturation and the second ones to hairpin melting.

Table S2. NMR assignments list of **IDJ1**, pH 7, T=5°C.

Residue	H1′	H2′	H2′′	H5/H2/M	H6/8	H41/21/61	H42/22/62	H1/H3
C1	5.97	2.04	2.32	5.88	7.84	9.78	8.35	no
C2	6.44	2.17	2.59	5.86	7.79	9.57	8.34	15.59
C3	6.24	1.10	2.07	5.92	7.53	9.79	7.92	15.39
G4	5.96	2.68	2.98	-	8.31	8.85	5.95	11.33
T5/30	6.09	2.07	2.31	1.76	7.64	-	-	na
T6/31	6.48	2.20	2.57	1.92	7.82	-	-	na
T7	6.48	2.54		2.06	7.96	-	-	11.93
C8	6.45	2.00	2.53	6.03	7.84	9.32	8.24	15.66
C9	6.19	2.06	2.26	5.79	7.62	9.15	8.19	15.54
T10	6.10	1.65	2.41	1.47	7.24	-	-	10.48
C11	5.31	2.03	2.36	5.78	7.45	8.88	7.05	-
G12	5.83	2.67	2.69	-	7.89	no	no	13.08
C13	5.60	1.90	2.31	5.36	7.27	8.41	6.51	-
G14	5.43	2.67	2.73	-	7.90	no	no	12.84
A15	5.94	2.35	2.67	7.60	7.94	7.89	6.05	-
A16	5.88	2.09	2.43	7.72	7.47	na	6.44	-
G17	5.35	2.35	2.67	-	8.11	no	no	no
C18	5.70	1.60	2.03	5.21	7.20	no	no	-
A19	5.69	3.04	3.37	8.11	8.04	no	no	-
T20	6.32	2.95	2.51	1.84	7.46	-	-	13.51
T21	6.12	2.23	2.54	1.63	7.47	-	-	13.99
C22	5.63	2.13	2.43	5.68	7.55	8.60	7.04	-
G23	5.86	2.64	2.73	-	7.93	no	no	12.95
C24	5.70	1.60	2.18	5.40	7.23	8.44	6.65	-
G25	5.92	2.65	2.82	-	7.94	no	no	13.04
C26	6.06	1.91	2.32	5.63	7.42	no	no	no
C27	6.32	2.02	2.52	5.82	7.63	9.07	8.08	15.59
C28	6.16	1.13	2.07	5.84	7.49	9.30	7.56	15.39
G29	5.95	2.68	2.98	-	8.30	8.86	5.95	11.11
T32	6.48	2.54		2.06	7.96	-	-	11.91
C33	6.49	2.11	2.55	6.06	7.85	9.49	8.26	15.66
C34	6.06	2.19	2.52	5.85	7.70	9.43	8.36	15.54
T35	6.42	2.33	2.46	1.74	7.35	-	-	11.48

no: not observed, na: not assigned

$H3^+$ of $C{:}C^+$ base-pair are shown in the two cytosines involved.

Table S3. Experimental constraints and calculation statistics of **IDJ1**.

Experimental distance constraints		
Total number	238	
Intra-residue	102	
Sequential	83	
Others	53	
Interphase	25	
I-motif	100	
Duplex	113	
RMSD (Å)		
I-motif region[1] bases	0.5±0.1	
Hairpin region[2] bases	1.1±0.4	
I-motif region[1] heavy atoms	1.0±0.3	
Hairpin region[2] heavy atoms	1.7±0.5	
All heavy atoms	3.3±1.0	
Violations	Average	Range
Sum of violations (Å)	1.37	1.07/1.74
Maximum violations (Å)	0.24	0.17/0.32
NOE energy (kcal/mol)	23.6	19.8/23.6
Total energy (kcal/mol)	-2095	-2483/-2094

[1] Residues 1-3,8,9,26-28,33,34

[2] Residues 11-25

[3] K_{NOE} = 20 kcal

Table S4. Average dihedral angles and order parameters of the structure of **IDJ1**.

Res	Pucker		α		β		γ		δ		ε		ζ		χ	
	P	A	M	OP	M	OP	M	OP	M	OP	M	OP	M	OP	M	OP
C1	28	38	-	-	-	-	57	1.0	80	1.0	-167	1.0	-	-	-134	1.0
C2	63	34	-61	1.0	-178	1.0	72	1.0	118	0.9	-170	1.0	-89	1.0	-122	1.0
C3	166	43	-64	0.6	172	1.0	94	0.6	149	1.0	-178	1.0	-99	0.9	-102	1.0
G4	172	37	-72	1.0	-175	1.0	59	1.0	148	1.0	-162	1.0	-92	1.0	-82	1.0
T5	173	37	-43	0.6	176	1.0	39	0.5	150	1.0	-104	1.0	158	1.0	-135	1.0
T6	173	39	-75	0.8	-180	0.9	71	0.9	151	1.0	-85	1.0	113	0.9	-144	1.0
T7	29	39	-129	0.6	-178	1.0	61	0.7	78	1.0	-165	1.0	66	1.0	-141	1.0
C8	151	34	-19	0.4	-174	1.0	24	0.5	137	1.0	-147	1.0	-97	1.0	-123	1.0
C9	137	50	-80	1.0	-174	1.0	55	1.0	132	1.0	-142	0.9	-88	1.0	-81	1.0
T10	148	44	-133	0.2	177	0.9	144	0.3	139	1.0	-172	1.0	-166	0.8	-103	1.0
C11	158	39	-81	0.8	151	0.7	100	0.6	139	1.0	-168	1.0	-88	1.0	-113	1.0
G12	160	41	-82	1.0	161	0.6	73	0.6	142	1.0	-158	1.0	-88	1.0	-113	0.9
C13	141	45	-89	1.0	150	0.6	69	0.6	132	1.0	-152	0.9	-80	1.0	-117	1.0
G14	164	40	-80	1.0	173	1.0	52	1.0	145	1.0	-166	0.9	-125	0.8	-108	1.0
A15	177	33	-68	1.0	-178	1.0	52	1.0	142	1.0	-155	1.0	-122	0.8	-104	1.0
A16	122	41	-93	1.0	173	1.0	50	1.0	121	0.9	-159	0.6	-89	1.0	-82	1.0
G17	157	40	102	0.3	-154	0.8	142	0.5	142	1.0	-143	0.9	-116	0.4	-61	0.5
C18	136	42	-85	0.9	167	0.9	50	0.9	131	0.9	-136	0.7	-117	0.7	-120	0.6
A19	159	46	1	0.3	169	0.7	146	0.7	147	1.0	-160	0.7	-4	0.2	-92	1.0
T20	163	36	-86	0.9	147	0.8	88	0.7	138	1.0	-162	1.0	-119	0.6	-126	1.0
T21	160	40	-94	0.8	136	0.6	113	0.5	143	1.0	-169	1.0	-87	1.0	-112	0.9
C22	157	40	-55	0.2	-158	0.7	98	0.5	139	1.0	-166	0.9	-119	0.7	-122	1.0
G23	158	39	-61	0.4	-174	1.0	72	0.5	138	1.0	-173	1.0	-90	1.0	-101	1.0
C24	146	45	-75	1.0	165	0.7	66	0.7	134	1.0	-154	1.0	-89	1.0	-121	1.0
G25	27	37	-145	1.0	176	1.0	59	1.0	82	1.0	-157	1.0	-87	1.0	-116	1.0
C26	131	41	-97	0.9	98	0.8	174	0.9	119	0.9	-134	1.0	-77	1.0	-136	1.0
C27	76	43	145	0.4	180	0.8	170	0.7	102	0.9	-158	1.0	-85	1.0	-115	1.0
C28	164	41	-162	0.4	148	0.7	-163	0.6	146	1.0	-175	1.0	-82	0.9	-102	1.0
G29	166	39	-74	0.6	178	1.0	73	0.7	146	1.0	-157	1.0	-87	1.0	-89	1.0
T30	173	35	-40	0.6	179	1.0	29	0.5	151	1.0	-112	1.0	157	1.0	-132	1.0
T31	182	41	-61	0.3	163	1.0	57	0.4	158	1.0	-76	1.0	82	1.0	-149	1.0
T32	20	40	69	1.0	-174	1.0	-178	1.0	79	1.0	-156	1.0	78	1.0	-144	1.0
C33	71	40	-75	0.8	-166	1.0	63	0.8	93	0.9	-159	0.8	-87	1.0	-129	1.0
C34	55	41	-94	0.4	175	0.9	103	0.6	86	1.0	-166	0.9	-79	0.8	-120	1.0
T35	146	42	-43	0.7	-165	0.9	58	0.6	137	1.0	-	-	-91	1.0	-102	1.0

Table S5. Average values of selected helical parameters of **IDJ1**.

Base par	Rise	Twist
A15-T21	3.1	38
G14-C22	3-0	37
C12-G23	3.1	26
G12-C24	2.7	32
C11-G25	3.1	27
T10-T35	3.5	-31
C1-C26	3.4	54
C9-C34	3.4	-34
C2-C27	3.0	51.
C8-C33	3.4	-32
C3-C28	2.9	54

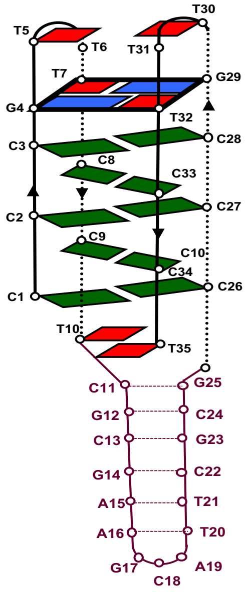

References

(1) Phan. A.T.; Patel. D.J. *J. Am. Chem. Soc*. **2002**, 124, 1160.

(2) Plateau. P.; Guéron. M. *J. Am. Chem. Soc.* **1982**, *104*, 7310.

(3) Kumar. A.; Ernst. R. R.; Wüthrich. K. *Biochem. Biophys. Res. Commun.* **1980**, *95*, 1.

(4) Bax. A.; Davis. D. G. *J. Magn. Reson.* **1985**, *65*, 355.

(5) Piotto. M.; Saudek. V.; Sklenář. V. *J. Biomol. NMR* **1992**, *2*, 661.

(6) Cai. L.; Chen. L.; Raghavan. S.; Rich. A.; Ratliff. R.; Moyzis. R. *Nucleic Acids Res.* **1998**, *26*, 4696.

(7) Güntert. P.; Mumenthaler. C.; Wüthrich. K. *J. Mol. Biol.* **1997**, *273*, 283.

(8) D.A. Case. I.Y. Ben-Shalom. S.R. Brozell. D.S. Cerutti. T.E. Cheatham. III. V.W.D. Cruzeiro. T.A. Darden. R.E. Duke. D. Ghoreishi. M.K. Gilson. H. Gohlke. A.W. Goetz. D. Greene. R Harris. N. Homeyer. Y. Huang. S. Izadi. A. Kovalenko. T. Kurtzman. T.S. Lee. S. LeGrand. P. Li. C. Lin. J. Liu. T. Luchko. R. Luo. D.J. Mermelstein. K.M. Merz. Y. Miao. G. Monard. C. Nguyen. H. Nguyen. I. Omelyan. A. Onufriev. F. Pan. R. Qi. D.R. Roe. A. Roitberg. C. Sagui. S. Schott-Verdugo. J. Shen. C.L. Simmerling. J. Smith. R. SalomonFerrer. J. Swails. R.C. Walker. J. Wang. H. Wei. R.M. Wolf. X. Wu. L. Xiao. D.M. York and P.A. Kollman (2018). AMBER **2018**. University of California. San Francisco.

(9) Soliva. R.; Monaco. V.; Gómez-Pinto. I.; Meeuwenoord. N. J.; Marel. G. a; Boom. J. H.; González. C.; Orozco. M. *Nucleic Acids Res.* **2001**, *29*, 2973.

(10) Ivani. I.; Dans. P. D.; Noy. A.; Pérez. A.; Faustino. I.; Hospital. A.; Walther. J.; Andrio. P.; Goñi. R.; Balaceanu. A.; Portella. G.; Battistini. F.; Gelpí. J. L.; González. C.; Vendruscolo. M.; Laughton. C. A.; Harris. S. A.; Case. D. A.; Orozco. M. *Nat. Methods* **2015**, *13*, 55.

(11) Koradi. R.; Billeter. M.; Wüthrich. K. *J. Mol. Graph.* **1996**, *14*, 51.

(12) Lu. X.; Olson. W. *Nat. Protoc.* **2008**, *3*, 1213.

(13) a) M. Cheng. D. Qiu. L. Tamon. E. Ištvánková. P. Víšková. S. Amrane. A. Guédin. J. Chen. L. Lacroix. H. Ju. L. Trantírek. A. B. *Angew. Chem. Int. Ed.* **2021,** *60*, 10286; b) A. M. Fleming. Y. Ding. R. A. Rogers. J. Zhu. J. Zhu. A. D. Burton. C. B. Carlisle and C. J. Burrows. *J. Am. Chem. Soc.* **2017**, *139*, 4682.
